# Intracellular Insulin-like growth factor binding protein 2 (IGFBP2) contributes to the senescence of keratinocytes in psoriasis by stabilizing cytoplasmic p21

**DOI:** 10.18632/aging.103045

**Published:** 2020-04-17

**Authors:** Laura Mercurio, Daniela Lulli, Francesca Mascia, Elena Dellambra, Claudia Scarponi, Martina Morelli, Carola Valente, Maria Luigia Carbone, Sabatino Pallotta, Giampiero Girolomoni, Cristina Albanesi, Saveria Pastore, Stefania Madonna

**Affiliations:** 1 Laboratory of Experimental Immunology, IDI-IRCCS, Fondazione Luigi M. Monti, Rome, Italy; 2 Laboratory of Molecular and Cellular Biology, IDI-IRCCS, Fondazione Luigi M. Monti, Rome, Italy; 3 V Dermatology Division, IDI-IRCCS, Fondazione Luigi M. Monti, Rome, Italy; 4 Department of Medicine, Section of Dermatology, University of Verona, Verona, Italy

**Keywords:** psoriasis, senescence, insulin-like growth factor binding protein 2, keratinocytes, p21CIP1/WAF1

## Abstract

Psoriasis is a chronic Th1/Th17 lymphocytes-mediated inflammatory skin disease, in which epidermal keratinocytes exhibit a peculiar senescent state, resistance to apoptosis and the acquisition of senescence-associated secretory phenotype (SASP). SASP consists of the release of soluble factors, including IGFBPs, that exert extracellular and intracellular functions in IGF-dependent or independent manner.

In this report, we investigated the expression and function of IGFBP2 in senescent keratinocytes isolated from the skin of patients with plaque psoriasis. We found that IGFBP2 is aberrantly expressed and released by these cells *in vivo*, as well as *in vitro* in keratinocyte cultures undergoing progressive senescence, and it associates with the cyclin-dependent kinase inhibitors p21 and p16 expression. For the first time, we provide evidence for a dual action of IGFBP2 in psoriatic keratinocytes during growth and senescence processes. While extracellular IGFBP2 counter-regulates IGF-induced keratinocyte hyper-proliferation, intracellular IGFBP2 inhibits apoptosis by interacting with p21 and protecting it from ubiquitin-dependent degradation. Indeed, we found that cytoplasmic p21 sustains anti-apoptotic processes, by inhibiting pro-caspase 3 cleavage and JNK phosphorylation in senescent psoriatic keratinocytes. As a consequence, abrogation of p21, as well as that of IGFBP2, found to stabilize cytoplasmic p21 levels, lead to the restoration of apoptosis mechanisms in psoriatic keratinocytes, commonly observed in healthy cells.

## INTRODUCTION

Psoriasis is an immune-mediated skin disease with a genetic predisposition, characterized by regional expansion and activation of T helper (Th)-1, Th-17, and Th-22 cells, releasing high levels of the pro-inflammatory cytokines IFN-γ, TNF-α, IL-17, and IL-22 in the skin [1, 2]. Epidermal keratinocytes respond to this potent pro-inflammatory environment by hyper-proliferating and releasing in turn numerous cytokines and chemokines, responsible for the recruitment and inflammatory auto-amplificatory loop in the skin [3]. In psoriatic plaques, hyper-proliferation of the basal keratinocytes is due to an increase of their mitotic activity, with 3 to 5 days required to move from basal to cornified layer instead of the normal 28 to 30 days [4]. This shortened maturation time is accompanied by defects of differentiation, with focal absence of the granular layer and parakeratosis [5]. In a unique fashion among chronic inflammatory skin disorders, psoriatic keratinocytes of the mid and upper epidermal layers undergo a sharp senescence switch, characterized by arrest of cell cycle and a peculiar resistance to apoptosis. Th1/Th17-released cytokines might contribute to the senescence state, by inducing anti-apoptotic programs in keratinocytes, and interfering with their terminal differentiation and cornification, both typical changes associated with the senescence state [6–8].

The senescence state is also strictly associated with a specific gene expression program that leads to massive production of soluble factors, known as the senescence-associated secretory phenotype (SASP), which affect the behaviour of resident and infiltrating cell populations, in autocrine and paracrine fashions [8, 9]. SASP includes inflammatory mediators, such as cytokines, chemokines and growth factors, but also regulatory or inhibitory factors, such as inhibitors of metalloproteinases and growth factor binding proteins, whose composition varies among the stages of senescence progression [10]. Among the regulatory factors of SASP, IGFBPs family, composed of six members (IGFBP 1-6), plays an important role in senescence and aging [7, 10, 11]. The better characterized function of IGFBPs is to regulate IGF-1 and -2 access to their receptors, and hence IGF bioactivity. IGF-driven signalling is involved in proliferation, survival and migration of epithelial cells [12], and its upregulation appears to be implicated in the pathogenesis of hyperplastic skin disorders, including psoriasis and non-melanoma skin cancers [13]. To date, most of reports described expression and function of IGFBP2 in a variety of cancers, where it is highly expressed and modulates the mitogenic IGF functions in the intercellular space. Other than binding and inhibiting IGF, IGFBP2 is known to exert IGF-independent extracellular and intracellular actions, most of which contributing to the arrest of cell growth, as described in transformed cells [14, 15]. To date, the expression, regulation and function of IGFBP2 in psoriasis remain unexplored.

At the molecular level, senescence is characterized by an enhanced expression of proteins involved in regulation of cell cycle, including cyclin-dependent kinase inhibitors p16INK4a (p16) and p21CIP1/WAF1 (p21) [16–19]. In particular, p16 binds to cyclin-dependent kinases (CDK)4/6 and abrogates their binding to cyclin D1, thereby determining cell cycle arrest in G1 phase. By contrast, p21 arrests cell cycle depending on its subcellular localization [20]. In the nucleus, p21 modulates the activity of transcription factors and serves as negative cell cycle regulator, whereas its cytoplasmic phosphorylated form is mainly involved in apoptosis inhibition, by inhibiting caspase 3 and the pro-apoptotic signal-regulating kinase 1 (ASK1) and c-Jun N-terminal kinase (JNK) [21, 22].

In this report, we investigated the expression and function of IGFBP2 in senescent keratinocytes isolated from the skin of patients with plaque psoriasis. We found that IGFBP2 is aberrantly expressed and released by these cells *in vivo*, as well as *in vitro* in keratinocyte cultures undergoing progressive senescence. For the first time, we provide evidence for a dual action of IGFBP2 in keratinocytes during growth and senescence processes. While extracellular IGFBP2 counter-regulates IGF-induced keratinocyte hyper-proliferation, intracellular IGFBP2 sustains the senescence and anti-apoptotic processes typical of psoriatic keratinocytes by stabilizing the cytoplasmic levels of p21.

## RESULTS

### IGFBP2 is upregulated in psoriatic keratinocytes and is closely associated with the cyclin-dependent kinase inhibitors p21 and p16

Keratinocyte cultures established from skin lesions of psoriatic patients are characterized by a rapid loss of the proliferative potential and a fast enrichment of p16^+^/ Ki67^-^ cells, thus denoting premature senescence-like changes [23, 24]. In line with these reports, a full transcriptome analysis performed by our group on psoriatic keratinocyte cultures confirmed a strong upregulation of a set of genes, including those encoding for p21, p16 and p57, implicated in the arrest of cell cycle and senescence switch, compared to cells obtained from healthy donors (unpublished data). Interestingly, among the mRNAs differentially expressed in psoriatic keratinocytes, IGFBP2, but not other IGFBP family members, was found to be significantly upregulated.

**Figure 1:**
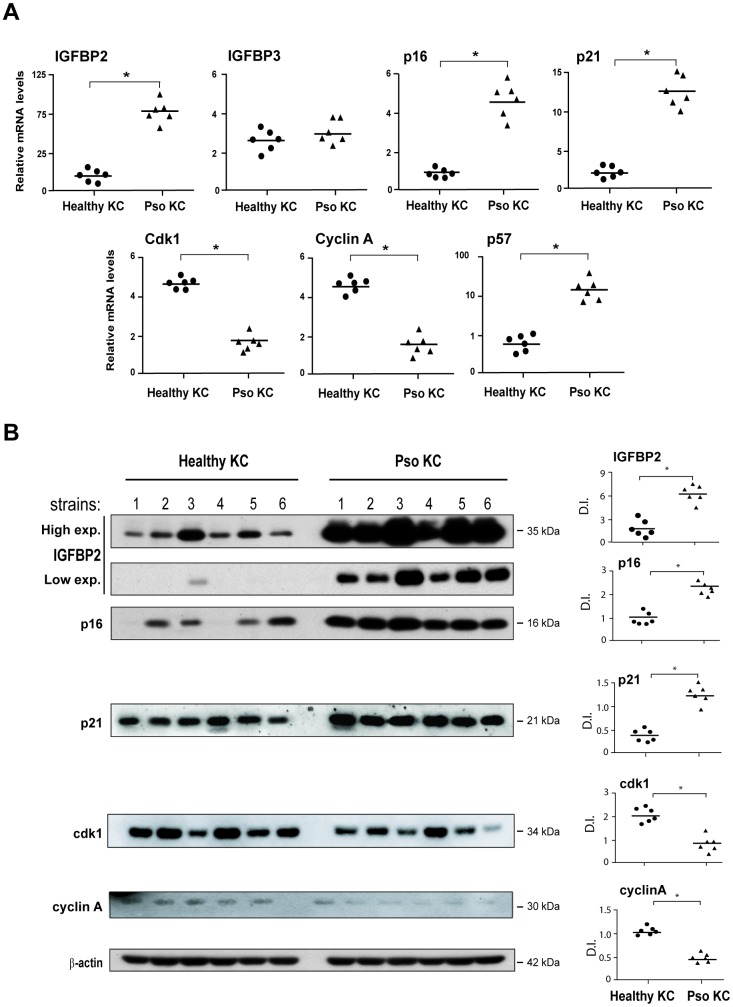
**Psoriatic keratinocyte cultures display enhanced IGFBP2 expression, together with an altered expression of genes implicated in the regulation and cell cycle arrest.** (**A**) Real-time PCR analysis was performed on keratinocyte cultures (at passage P4), obtained from lesional skin of psoriatic patients (*n* = 6) (pso KC) and healthy volunteers (*n* = 6) (healthy KC). Results are shown as individual values of relative mRNA levels (normalized to β-actin) of IGFBP2, IGFBP3, p16, p21 Cdk1, cyclin A and p57 and means of the two different groups. (**B**) WB analysis was performed on protein lysates from keratinocyte cultures isolated from healthy (*n* = 6) and lesional skin (*n* = 6) by using anti-IGFBP2, cyclin A, cdk1, -p16 and -p21 Abs. β-actin was used as loading control. Bands relative to IGFBP2 were showed at two different exposure times (High exp. 1 min; low exp., 30 seconds). Graphs represent the individual values and the means of the densitometric intensity (D.I.) of each band. (**A**, **B**), *p ≤ 0.05, as calculated by the Mann–Whitney U test.

To validate transcriptome data, we firstly performed Real-time PCR analysis on different strains of keratinocytes isolated from lesional (LS) skin biopsies of psoriatic patients (pso KC), as well as on cells obtained from healthy donors (healthy KC). Notably, as shown in Figure 1A, pso KC displayed higher mRNA levels of the senescent markers p16, p21 and p57, compared to healthy KC, whereas mRNA levels of Cdk1 and cyclin A, which promote the progression of cell cycle and cellular proliferation, were consistently down-regulated in pso KC (Figure 1A).

In line with gene expression data, pso KC showed higher mRNA levels of IGFBP2, but not of the other IGFBP members, including IGFBP3, compared to healthy cells (Figure 1A). In keeping with the IGFBP2 transcript data, IGFBP2 protein was found upregulated in different strains of pso KC, whereas a weaker expression of IGFBP2 was observed in healthy cell lysates (Figure 1B). Similarly, p16 and p21 protein expression was higher in pso KC strains than in healthy KC, whereas cyclin A and cdk1 levels were consistently lower in affected cells (Figure 1B).

Taken together, these findings unveiled a peculiar enhanced expression of intracellular IGFBP2 in psoriatic keratinocytes, together with that of other senescence markers and the down-regulation of proliferation markers. This suggests a potential involvement of IGFBP2 in cell cycle arrest and senescence of keratinocytes of psoriasis lesions.

### IGFBP2 is highly expressed *in vivo* in the senescent keratinocyte compartment of psoriatic skin lesions, and is induced *in vitro* by psoriasis-related cytokines

**Figure 2:**
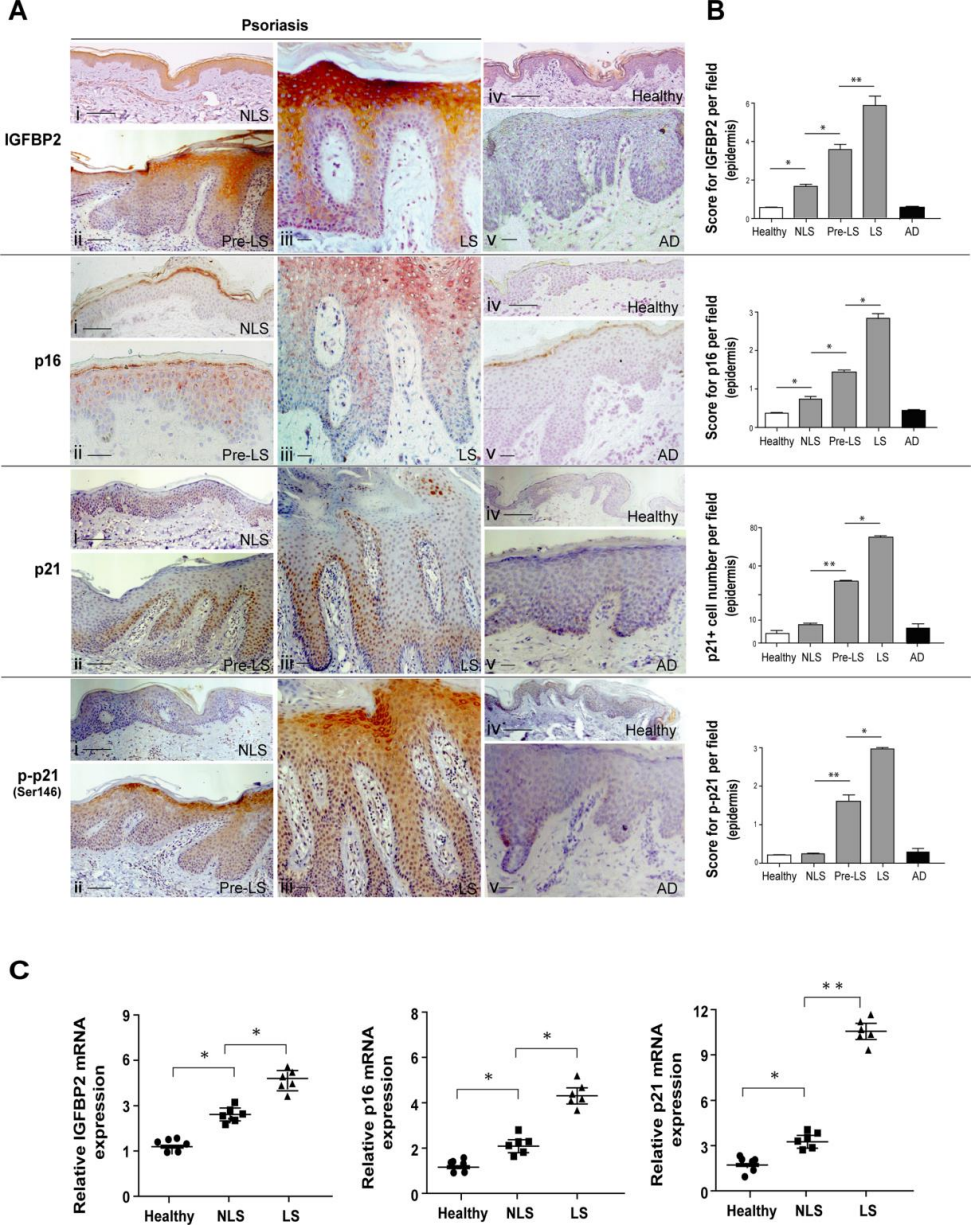
**IGFBP2 is enhanced in the suprabasal layers of lesional psoriatic skin, and parallels p16 and p21 senescence markers.** (**A**) IHC analysis of IGFBP2, p21 and phosphorylated p21 (p-p21, red-brown stained), as well as of p16 (red-stained), was performed on paraffin-embedded sections of biopsies obtained from psoriatic skin (*n* = 10), including non-lesional (NLS) (**i**), proximal-to-lesion (Pre-LS) (**ii**) and lesional (LS) zones of evolving plaques (**iii**), as well as from healthy donors (*n* = 6) (**iv**) and AD skin (*n* = 6) (**v**). Sections were counterstained with Mayer’s H&E. Bars, 100 μm. (**B**) Graphs show the mean of four-stage score values for IGFBP2, p16 and p-p21 epidermal expression, or the mean of the number of p21-positive cells ± SD. Two different sections were analysed for each staining, and the positivity was evaluated in five adjacent fields. (**C**) mRNA expression of IGFBP2, p16 and p21 was analysed by Real-time PCR on total RNA from healthy, NLS, LS biopsies (*n* = 6) and normalized to GAPDH levels. The results are shown as individual values, mean and ± SD of relative mRNA levels. In (**B**, **C**), *p ≤ 0.05, p** ≤ 0.01, as calculated by Mann–Whitney U test.

IGFBP2 expression was evaluated *in vivo* in biopsies of LS, proximal-to-lesion (Pre-LS) and non lesional (NLS) skin of psoriatic patients. In all the biopsies examined, IGFBP2 progressively increased from the adjacent Pre-LS (ii) to the LS area within the same skin biopsy (iii), with stronger staining in the suprabasal layers and reaching the highest intensity in the subcorneal zone (iii) (Figure 2A). In particular, the enhanced IGFBP2 expression was found to be concentrated in the compartment of psoriatic plaque enriched in senescent keratinocytes, suggesting a potential role specifically in this epidermal district. On the contrary, IGFBP2 expression was weak in NLS skin distant from lesions (i), and even totally absent in the epidermis of healthy skin (iv), or in lesions of AD patients (v). A similar expression pattern was observed for p16 (Figure 2A), with some positivity in the upper epidermal layers of Pre-LS skin (ii) and very strong expression in the spinous and granular epidermis layers of psoriatic lesions (iii), thus confirming the senescence state of keratinocytes located within these districts. Consistent with IGFBP2, p16 expression was weakly detectable in the epidermis of distant NLS of psoriatic patients (i), and absent in healthy controls (iv) or in the skin of AD patients (v). In line with a recent *in vivo* study [19], we found that the number of p21 positive cells progressively increased through the transition from Pre-LS (Figure 2A, ii) to LS (iii) skin, where p21 localization was mainly observed in the nucleus of keratinocytes of the spinous layers of epidermis (Figure 2A). p21 staining was undetectable in healthy epidermis (iv), and weakly detected in a low number of keratinocytes within NLS (i) and AD (v) epidermis. Of interest, phosphorylated p21 (p-p21) expression was progressively upregulated from Pre-LS to LS area of the same biopsies, but with a predominant distribution in the cytoplasm of psoriatic keratinocytes within subcorneal epidermis layers, similarly to what observed with IGFBP2 (Figure 2A, ii, iii). In contrast, p-p21 staining was quite absent in healthy (iv) and NLS epidermis (i), as well as in AD lesion (v). Accordingly, mRNA levels of IGFBP2, as well as those of p16 and p21, were significantly higher in the whole LS biopsies, compared to those detected in NLS samples (Figure 2C). In line with immunohistochemistry analyses, the transcriptional levels of IGFBP2, p16 and p21 were higher in NLS biopsies, when compared to healthy skin biopsies (Figure 2C).

We further investigated the expression and regulation of IGFBP2, p16 and p21 in keratinocytes isolated from NLS areas, left untreated or treated with combinations of psoriasis-related pro-inflammatory cytokines. As shown in Supplementary Figure 1, IFN-γ and TNF-α induced IGFBP2 and p16, both at transcriptional and protein level, and this effect was more pronounced when these cytokines were administrated together with IL-17A and IL-22 (Supplementary Figure 1A, 1B). Similarly, the basal and phosphorylated forms of p21 protein were induced by IFN-γ and TNF-α, and, at higher extent, by their combination with IL-17A and IL-22, although p21 transcriptional levels were not affected by these cytokines (Supplementary Figure 1A, 1B).

Hence, IGFBP2 is upregulated in the senescent keratinocyte compartment of the psoriatic skin lesions, characterized by a strong expression of p16, and its distribution is closely associated with that of the phosphorylated p21.

### Extracellular IGFBP2 is induced by IGF-1 and is functionally active in psoriatic keratinocytes

**Figure 3:**
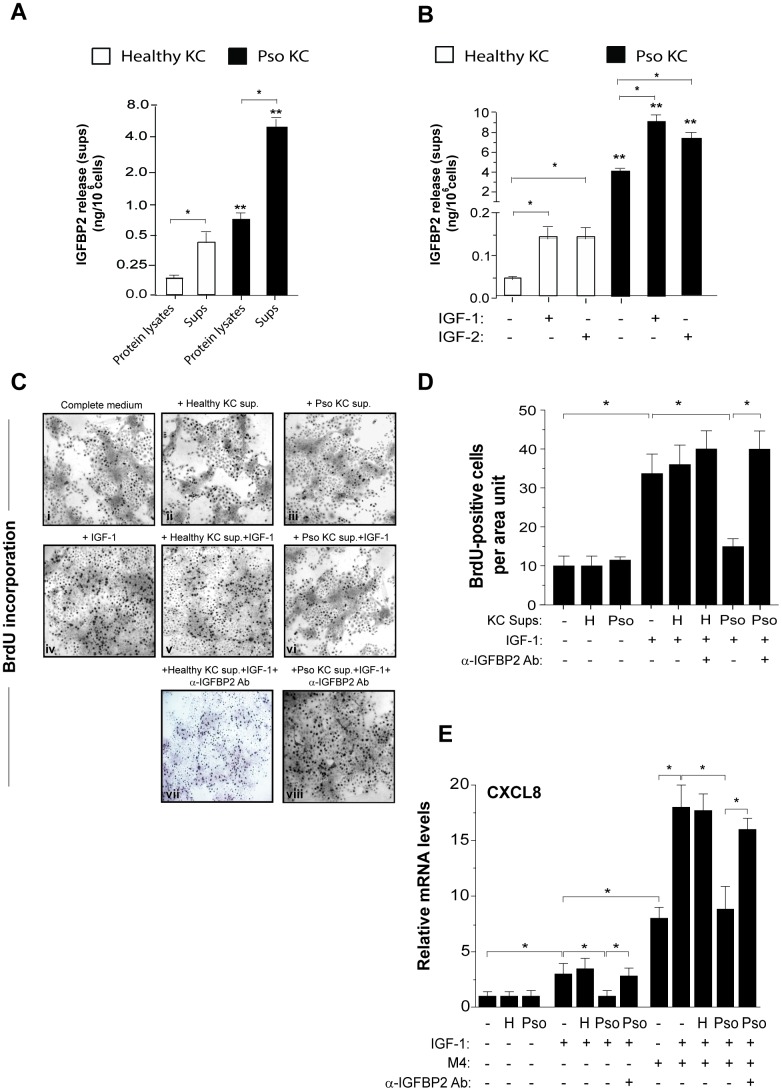
**IGFBP2 production is induced by IGF-1 and is functionally active in psoriatic keratinocytes.** IGFBP2 release was analysed by ELISA in protein lysates and supernatants (sups) of untreated healthy and pso KC cultures (**A**) or in sups of healthy and pso KC stimulated or not with 10 ng/ml IGF-1 or IGF-2 for 24 hours. (**B**) Data are expressed as mean of ng/10^6^ cells ± SD of three different experiments carried out on different strains (*n* = 3). *p ≤ 0.05, as assessed by unpaired Student’s *t* test; **p ≤ 0.01, as calculated by the Mann–Whitney U test, comparing IGFBP2 production between healthy and pso KC groups. (**C**) BrdU incorporation was evaluated in healthy KC grown on coverslips and treated with complete medium alone (**i**) or 10-fold concentrated sup. of healthy KC (**ii**) or of pso KC (Pso) (**iii**). In parallel, healthy KC were treated with IGF-1 (10 ng/ml) alone (**iv**), or in presence of 10-fold concentrated healthy KC sups (**v**), or pso KC sups (**vi**). In two experimental conditions, 1 μg/ml of neutralizing anti-IGFBP2 Ab (α-IGFBP2) was added (**vii**, **viii**). Images are relative to one of three independent experiments performed on three different healthy KC strains. (**D**) Graphs represent the number of BrdU-positive cells counted in high power fields and expressed as cells per area unit ± SD (*n* [microscopic fields per slide] = 6); H, healthy KC sups; Pso, psoriatic KC sups. (**E**) CXCL8 mRNA levels were detected by Real-time PCR in healthy KC stimulated for 8 hours with IGF-1 (10 ng/ml) alone or in combination with M4, including IFN-γ (200 U/ml), TNF-α (50 ng/ml), IL-17A (50 ng/ml) and IL-22 (50 ng/ml), in presence or not of healthy (H) or psoriatic (Pso) supernatants. In two experimental settings, 1 μg/ml of neutralizing anti-IGFBP2 Ab (α-IGFBP2) was added. Data shown are means of relative mRNA expression (normalized to GAPDH) of three independent experiments. (**D**, **E**), *p ≤ 0.05, as calculated by unpaired Student’s *t* test.

Data shown in Figure 1 demonstrated an intracellular accumulation of IGFBP2 in psoriatic keratinocytes compared to healthy cells. However, we found that IGFBP2 was mostly released extracellularly by both psoriatic and healthy cells, reaching an amount of 4.4 ± 0.3 ng/ml in sups of pso KC *versus* 0.7 ± 0.3 ng/ml observed in healthy KC cultures (Figure 3A).

A variety of factors, including IGF system, are known to regulate the expression of IGFBP members [15]. Accordingly, both pso KC and healthy KC responded to exogenous IGF-1, as well as to IGF-2, with a significant increase of IGFBP2 release, despite of their different basal levels, as shown in Figure 3B. However, human keratinocytes are unable to express these two growth factors, as previously described [25]. In order to assess whether IGFBP2 released by psoriatic keratinocytes was functionally active, and hence able to bind and regulate IGF-1 binding to its receptors on cell surface, we collected sups from unstimulated cultures of psoriatic keratinocytes (pso KC, IGFBP2 concentration: 10.06 ± 1.20 ng/ml) and from healthy cells (IGFBP2 concentration: 0.06 ± 0.01 ng/ml). The supernatant was enriched of IGFBP2 by 10-fold concentration, and finally used in 1:2 dilution in functional assays on normal human keratinocytes. The final IGFBP2 concentration in the medium conditioned by sups of pso KC was 48.0 ± 15.0 ng/ml, whereas it was of 0.25 ± 0.2 ng/ml in medium conditioned by healthy KC. When compared to complete culture medium (Figure 3C, i), no macroscopic changes or perturbation of BrdU incorporation could be observed in healthy keratinocytes grown for 24 hours in the medium conditioned by healthy KC or pso KC (Figure 3C, ii or iii). By contrast, the medium conditioned by pso KC (Figure 3C, vi), but not by healthy KC (Figure 3C, v), impaired the proliferative response to exogenous IGF-1 (Figure 3C, iv), an activity that could be in turn reverted by addition of a neutralizing anti-IGFBP2 Ab (Figure 3C, viii). We did not observe a rescue of IGFBP2 Ab with sups of healthy KC, thus confirming the low levels of IGFBP2 in healthy cells (Figure 3C, vii). The results of BrdU incorporation, obtained from different strains of healthy or pso KC, are graphed and summarized in Figure 3D. Similarly, we found that the mRNA levels of CXCL8 were upregulated by IGF-1 alone and, at higher extent, by its combination with TNF-α, IFN-γ, IL-17A and IL-22 cytokines (M4) (Figure 3E). Of note, transcriptional CXCL8 expression was significantly impaired by sups of pso KC, but not of healthy KC, and it was rescued following the specific neutralization of IGFBP2 (Figure 3E).

Taken together, these data demonstrated that extracellular IGFBP2 is functionally active in inhibiting the direct and indirect pro-proliferative action of IGFs in psoriatic keratinocytes.

### IGFBP2 expression is associated with the senescence switch of psoriatic keratinocytes

**Figure 4:**
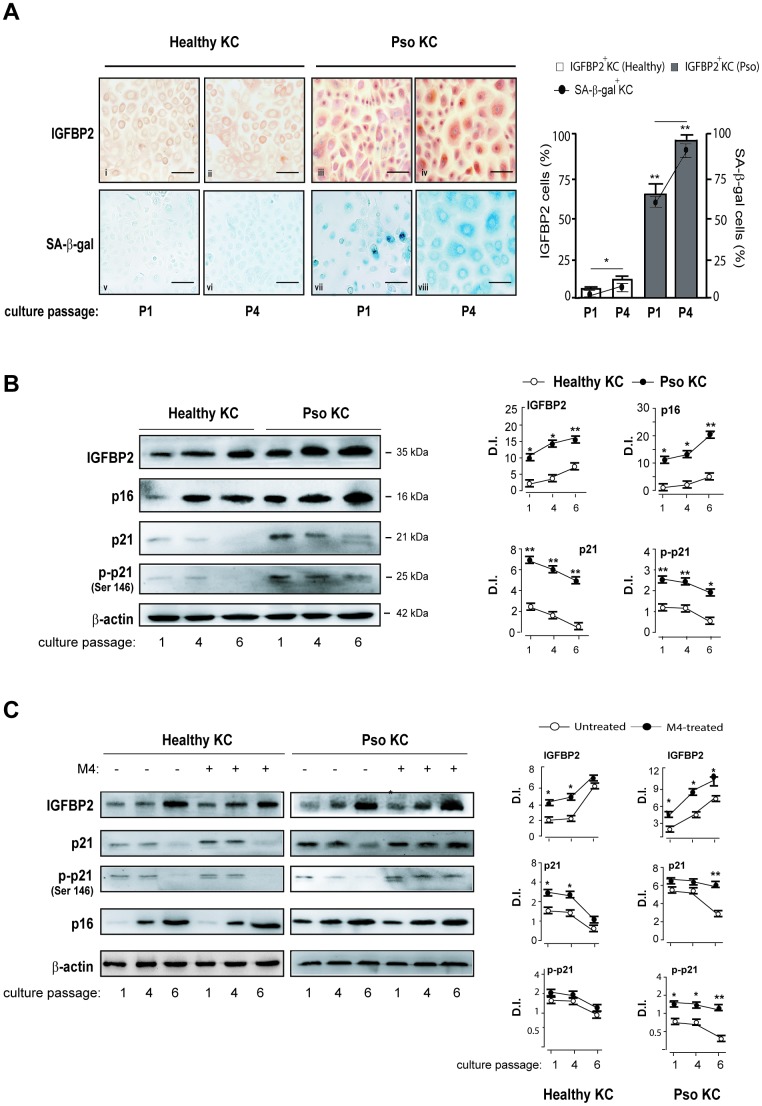
**IGFBP2 expression progressively increases during senescence changes in psoriatic KC.** (**A**) Healthy and pso KC were serially subcultured until they undergo senescence, and analysed by immunocytochemistry for the expression of IGFBP2 at two distinct culture passages (P1 and P4) (upper panels). Healthy KC were not counterstained with haematoxylin in order to preserve faint IGFBP2-specific staining (upper panels). The activity of senescence-associated β-galactosidase (SA- β-gal) was detected by colorimetric staining (blue) in healthy and pso KC cultures at passage P1 and P4. Data are representative of three independent experiments performed on different healthy (*n* = 3) and psoriatic (*n* = 3) donors. Bars, 50 μm. The graph shows the means of the percentage of IGFBP2 positive cells or SA- β-gal positive cells ± SD, counted in two adjacent fields. (**B**, **C**) WB analysis was performed on total protein lysates from healthy and pso KC at different serial passages of culture, left untreated (**B**) or M4-treated (**C**), to detect IGFBP2, p16, p21 and p-p21 expression. β-actin was used as loading control. Graphs represent the means of the densitometric intensity (D.I.) ± SD of the bands obtained from three different WB experiments. *p ≤ 0.05, **p ≤ 0.01, as calculated by unpaired Student’s t test, comparing healthy and pso KC groups, or M4-treated and untreated groups.

Data shown in Figures 1 and 2 suggested the involvement of the intracellular IGFBP2 in the senescence switch of psoriatic keratinocytes. To better investigate on IGFBP2 expression during senescence of keratinocytes, different strains of psoriatic and healthy keratinocytes were serially sub-cultured until they became senescent and subjected to immunocytochemistry analysis to detect the expression and distribution of IGFBP2 at two distinct culture passages (P1 and P4), likely resembling a pre-senescent and a highly senescent stage, respectively. The same cell strains were in parallel analysed in terms of senescence-associated β-galactosidase (SA-β-gal) activity, known to be peculiarly high in senescent cells, but not in quiescent or immortal cells [9]. As shown in Figure 4A, a high percentage of IGFBP2 positive cells were present in pso KC at culture passage P1, with a nuclear localization of IGFBP2 in small, proliferating cells, and a cytoplasmic distribution in very large, senescent cells, present at P1 of affected cells (iii). The number of IGFBP2 positive cells enhanced at passage P4, characterized by a high percentage of senescent cells, where its staining was mainly detectable in the cytoplasmic compartment (Figure 4A, iv). In contrast, IGFBP2 staining was faint in healthy KC at passage P1 (Figure 4A, i), whereas it was only slightly detectable in the cytoplasmic compartment at P4 (ii). In support with these findings, the blue, granule-shaped lysosome-restricted SA-β-Gal staining identified a subpopulation of large, elongated cells already present at P1 of pso KC (vii), which became highly represented at P4 (viii) and appeared to be positive to IGFBP2 staining (Figure 4A, iv). This subpopulation was absent in healthy KC, where a very weak SA-β-Gal staining was detectable in the cytoplasmic compartment of cells at the passage P4 only (Figure 4A, v and vi). We performed Western blotting analysis on psoriatic and healthy keratinocytes at different culture passages to investigate the involvement of IGFBP2 in the senescence switch. Notably, we found that healthy KC expressed increasing levels of p16, as well as progressively diminished levels of p21 and its phosphorylated form (p-p21), during serial culture passages (Figure 4B). Of note, these cells showed escalating levels of IGFBP2 protein, reaching the highest amount at P6, whereas IGFBP3 tended to decrease through culture passages (Figure 4B). Similarly, pso KC serially subcultured showed massive and increasing levels of IGFBP2, which were closely associated with increasing levels of p16 during the serial passages (Figure 4B). We found that, although p21 and p-p21 expression tended to decrease in pso KC during senescence, its levels remained markedly higher at all culture passages, compared to those observed in healthy KC (Figure 4B).

Furthermore, we analysed IGFBP2, p21 and p16 expression in healthy and psoriatic KC activated by cytokines (M4) during senescence progression. We found that p21, in both unphosphorylated and phosphorylated forms, remained high at passage 6 in M4-treated pso KC, compared to untreated cultures at the same passage. Here, IGFBP2 expression was also strongly induced by M4 treatment and this was evident both at passage 4 and 6 in pso KC. Contrarily, the levels of p21 in healthy KC decreased not only in untreated cultures, but also in M4-treated cells, where IGFBP2 expression was slightly induced at passage 1 and 4.

As a whole, these data revealed a strong association between IGFBP2 expression and senescence switch in psoriatic keratinocytes.

### IGFBP2 expression is positively regulated by p16 and it co-localizes with p16 and p21 in the cytoplasmic compartment of psoriatic keratinocytes

**Figure 5:**
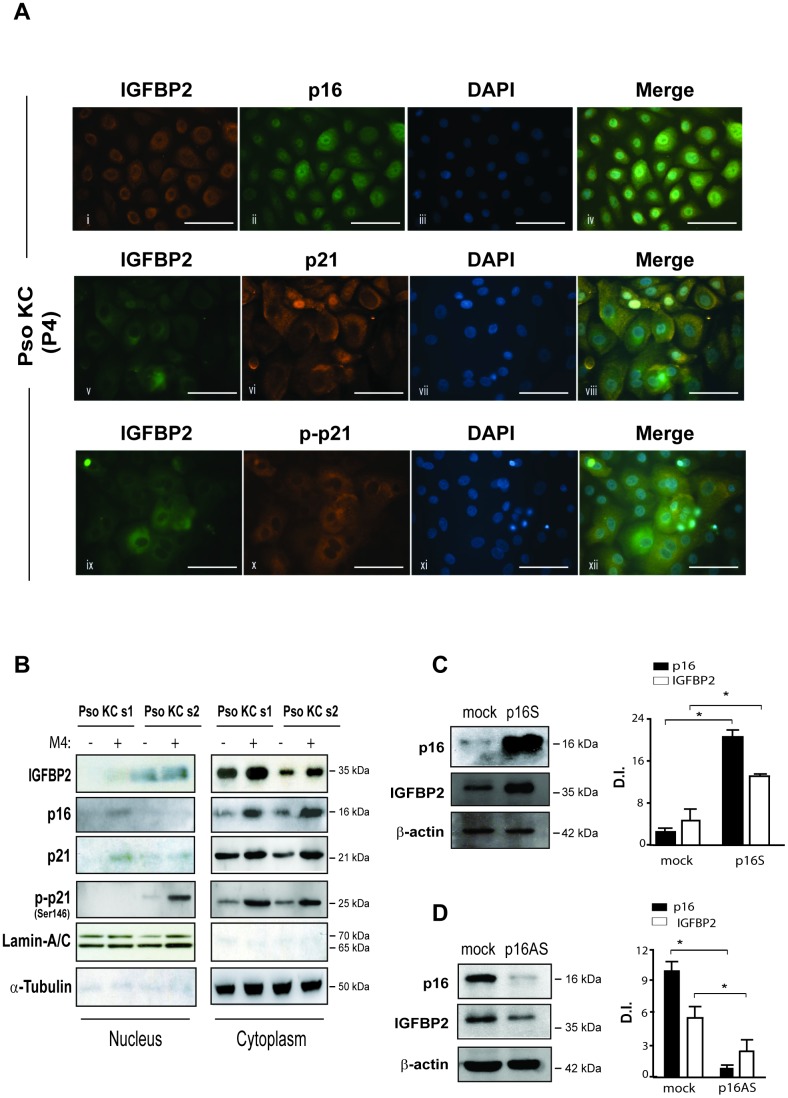
**IGFBP2 co-localizes with p21 in the cytoplasm of senescent psoriatic keratinocytes and its expression is positively regulated by p16.** (**A**) Immunofluorescence analysis was conducted on pso KC cultures (*n* = 3) at passage 4 (P4) to evaluate IGFBP2, p16, p21 and p-p21 subcellular localization. Cells were immunostained with anti-IGFBP2 Ab, followed by Cy3-conjugated secondary Ab (orange, panel i) or by Alexa Fluor 488-conjugated secondary Ab (green, panel v, ix), or, alternatively with p16 followed by Alexa Fluor 488 secondary Ab (green, panel ii), and p21 or p-p21 primary antibodies followed by Alexa Fluor 555 secondary Ab (orange, panels vi, x). Nuclei were counterstained with DAPI (blue) and the merging of three patterns was shown within the same field (merge, panels iv, viii, xii). Bars, 100 μM (**B**) IGFBP2, p16, p21 and p-p21 expression was analysed by WB on nuclear and cytosolic protein fractions obtained from two different pso KC strains (pso KC s1; pso KC s2), left untreated or treated with M4 for 18 hours. The quality of nuclear and cytosolic fractions was assessed by detection of lamin A/C and α-tubulin, respectively. (**C**) WB analysis was performed on lysates from primary human KC transduced with empty vector (mock) or p16 sense (p16S) vector and analysed for p16 and IGFBP2 expression. (**D**) Similarly, p16 and IGFBP2 expression was evaluated by WB on protein lysates obtained from primary KC cultures transduced with empty (mock) or antisense (p16AS). In (**C**, **D**), graphs show the means of the densitometric intensity (D.I.) of the bands ± SD, obtained from three independent experiments. *p ≤ 0.05, as calculated by Mann–Whitney U test.

To better define the expression and distribution of IGFBP2, as well as of the senescence markers p16 and p21, in psoriatic keratinocytes, we performed immunofluorescence analysis on pso KC cultured at P4 passage (senescent cells), characterized by a high number of senescent cells. In line with immunocytochemistry results, IGFBP2 was mainly expressed in the cytoplasm of very large, senescent pso KC, with a perinuclear intensification of the signal particularly evident in large, overtly senescent cells (Figure 5A, i, v and ix). However, small, proliferating psoriatic keratinocytes, poorly represented at culture P4, displayed a nuclear IGFBP2 distribution (Figure 5A, v). These findings are in line with recent reports, demonstrating the presence of a classical nuclear localization signal sequence in IGFBP2, responsible for its translocation into the nucleus of several cell types [26]. We observed a cytoplasmic co-expression of IGFBP2 and p16 in senescent subpopulation of pso KC (Figure 5A, i-iv). Notably, IGFBP2 co-localized with p21 in the cytoplasm of very large, senescent cells (Figure 5A, v-viii), as well as with phosphorylated form of p21, which was mainly localized in the cytoplasm of the senescent subpopulation, highly represented at passage P4 of pso KC cultures (Figure 5A, ix-xii).

Consistent with the immunofluorescence findings, Western blotting analysis revealed the accumulation of IGFBP2 protein, as well as of p16 and p21, in cytoplasmic fractions of untreated or cytokine-treated pso KC (Figure 5B). In line with data shown in Supplementary Figure 1, M4 stimulation induced the cytoplasmic levels of IGFBP2, as well as of p16 and p21 (in both basal and phosphorylated forms), in pso KC at culture P4 (Figure 5B). In light of the association of IGFBP2 with p16 during senescence progression (Figure 4B), we investigated whether p16 modulation could affect IGFBP2 expression in human keratinocytes. As shown in Figure 5C, the overexpression of full-length p16 in primary cultures of normal keratinocytes (p16S) at passage P1 (pre-senescent state) resulted in a significant increase of IGFBP2 levels, compared to control (mock) cells. Consistently, p16 down-regulation obtained by transducing primary keratinocytes cultured at passage P4 (senescent state) with an anti-sense p16 (p16AS) led to a significant decrease of IGFBP2 protein levels (Figure 5D).

As a whole, these data demonstrated that IGFBP2 expression is positively regulated by p16 and it co-localizes with p16 and p21 in the cytoplasmic compartment of psoriatic keratinocytes.

### IGFBP2 contributes to the arrest of proliferation and to the senescence of psoriatic keratinocytes by interacting with p21 and protecting it from ubiquitin-mediated proteasome degradation

**Figure 6:**
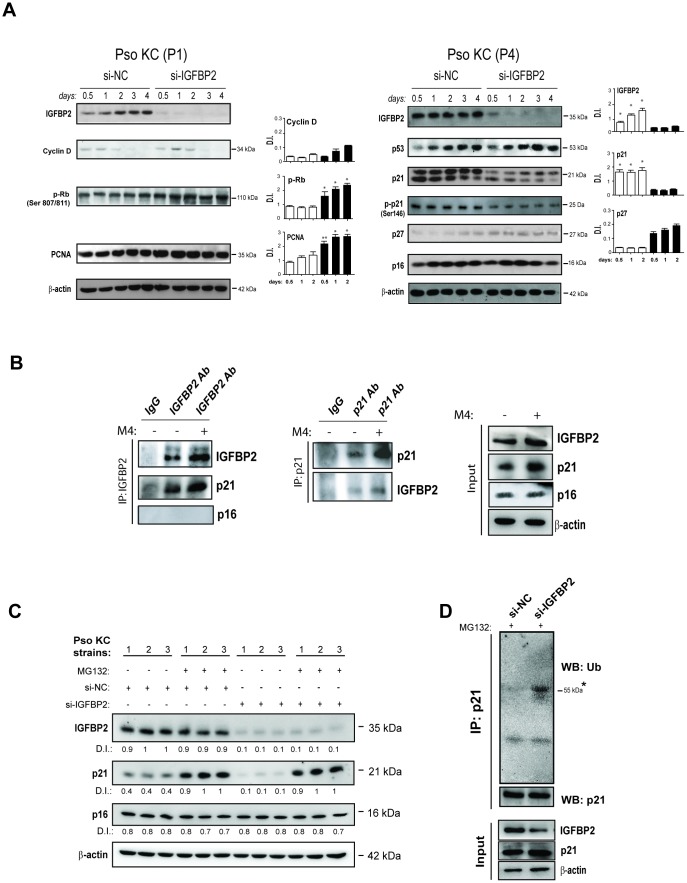
**IGFBP2 interacts with p21 and protects it from ubiquitin-mediated proteasome degradation.** (**A**) IGFBP2, p53, p21, p-p21, p27 and p16 protein expression was detected by WB analysis in pso KC cultured at passage 4 (P4, senescent cells) and silenced (si-IGFBP2) or not for IGFBP2 (si-NC) for different time points (right panels). Similarly, cyclin D, p-Rb and PCNA protein expression was detected in pso KC cultured at passage 1 (P1, pre-senescent cells) and silenced or not for IGFBP2 (left panels). In A and B, graphs show the mean ± SD of densitometric intensity (D.I.) of three independent experiments. *p ≤ 0.05, **p ≤ 0.01 as calculated by paired Student’s *t* test comparing si-IGFBP2 with si-NC. (**B**) Co-immunoprecipitation experiments were performed on protein lysates obtained from pso KC left untreated or treated by M4 for 6 hours and then immunoprecipitated with antibodies against IGFBP2 or goat IgG as negative control (IP: IGFBP2, left panel), and with p21 or mouse IgG (IP: p21, right panel). The immunoprecipitates were probed with anti-IGFBP2, -p21 or –p16 antibodies, as shown in left and right panels. WB analysis was also performed on cell lysates (Input) to detect IGFBP2 and p21 levels. Figures are representative of three independent experiments. (**C**) Protein extracts of three distinct pso KC strains, transfected with si-IGFBP2 or si-NC for 24 h and then treated or not with 20 μM of the proteasome inhibitor MG132 for 6 h, were subjected to WB for the detection of IGFBP2, p21 and p16 expression. WB panels are representative of three independent experiments and D.I. indicates values of densitometric intensity. (**D**) Endogenous p21 was immunoprecipitated by protein lysates obtained from pso KC cultures silenced or not for IGFBP2 for 24 h and treated with 20 μM of MG132 for 6 hours. WB analysis was performed for the detection of p21 ubiquitination by using anti-ubiquitin antibody. WB was also performed on cell lysates (Input) to detect IGFBP2 and p21 levels, as well as β-actin as loading control.

To investigate the molecular impact of IGFBP2 on the senescent state of psoriatic keratinocytes, the effects of IGFBP2 abrogation in pso KC cultured at passage P4 (senescent cells) were examined. As shown in Figure 6A, pso KC cultures silenced for IGFBP2 (si-IGFBP2) showed a significant strong decrease of p21 only at the protein levels, together with a reduction of its phosphorylated form (p-p21) at all transfection times, as compared to control cells (Figure 6A, right panels). Indeed, p21 mRNA levels are not affected by IGFBP2 knock-down (Supplementary Figure 2). Consistently, IGFBP2 knock-down determined a reduced β-galactosidase activity in both untreated and M4-treated senescent pso KC cultures (Supplementary Figure 3, panel B). Interestingly, p16 and also p53, the major transcriptional regulator of p21, remained unaltered in pso KC when IGFBP2 was silenced (Figure 6A, right panels). Consistent with reduced levels of p21 in senescent cells, IGFBP2-silenced pso KC cultured at passage 1 (pre-senescent cells) showed higher protein levels of PCNA, p-Rb, and cyclin-D, which all together promote cell cycle progression and cellular proliferation (Figure 6A, left panels). In line with these results, cell proliferation evaluated by using Trypan blue exclusion assay, resulted in an increased number of viable cells in IGFBP2-silenced pre-senescent pso KC compared with control cells, at all transfection (Supplementary Figure 3, panel A). However, a slight upregulation of p27, another member of Cdk-interacting protein/kinase-inhibitory protein (Cip/Kip) family, was visible in IGFBP2-silenced cells, plausibly in the context of a compensatory response (Figure 6A, right panels). In contrast to what observed with IGFBP2 interference, no significant change in cell morphology or detectable perturbation of cell proliferation (PCNA) or senescence markers (p16 and p21) could be observed in healthy keratinocytes over-expressing exogenous IGFBP2, suggesting that IGFBP2 alone is not sufficient to induce any detectable senescence change in normal human keratinocytes (Supplementary Figure 4).

Since IGFBP2 abrogation affected p21 protein amount, but not its transcriptional levels, we hypothesize that IGFBP2 could interact with p21 and stabilize it. Indeed, a previous study reported that IGFBP2 is capable of binding to p21 in growth-arrested alveolar lung cells [27]. To investigate on the possible interaction of IGFBP2 and p21 in psoriatic keratinocytes, co-immunoprecipitation experiments were performed on protein lysates obtained from pso KC, left untreated or treated with M4 cytokine stimulus. In particular, IGFBP2 and p21 were separately immunoprecipitated from cell lysates and the two proteins were reciprocally detected by Western blotting analysis. As shown in Figure 6B (left panels), a band corresponding to p21 was evident in untreated cell lysates immunoprecipitated for IGFBP2, the intensity of which increased in M4-treated lysates. Similarly, endogenous IGFBP2 was detected in p21 immunoprecipitated lysates, with the signal intensity being increased in M4-treated lysates (Figure 6B, right panels). In both co-immunoprecipitation experiments, IGFBP2 or p21 proteins were not detected in isotype IgG complexes, used as negative control (Figure 6B). Finally, no complexes between p16 and IGFBP2 could be immunoprecipitated (Figure 6B).

Taken together, these data demonstrated that IGFBP2 was able to bind p21 and positively regulate its protein level. As p21 is a labile protein rapidly degraded by the proteasome, we surmised that IGFBP2 might affect its stability. Indeed, MG132, a specific proteasome inhibitor, substantially rescued the decline of p21 protein caused by IGFBP2 knock-down in different strains of pso KC (Figure 6C), suggesting that IGFBP2 protects p21 from proteasomal degradation. As expected, the levels of p16 were not influenced neither by IGFBP2 silencing or MG132 treatment (Figure 6C).

p21 can be degraded by proteasome in ubiquitin-dependent or -independent manners [28]. To investigate the mechanism by which IGFBP2 protects p21 by proteasome degradation, endogenous p21 was immunoprecipitated from protein lysates of IGFBP2-silenced pso KC or control cells treated with MG132 inhibitor, and then subjected to Western blotting analysis for the detection of its ubiquitination state. As shown in Figure 6D, silencing of IGFBP2 expression led to a significant increase of endogenous p21 ubiquitination, compared to control cells.

These results clearly demonstrated that IGFBP2 interacts with p21 in the senescent psoriatic keratinocytes and protects it from ubiquitin-mediated proteasome degradation.

### IGFBP2 contributes to the arrest of proliferation and resistance to apoptosis of psoriatic keratinocytes

**Figure 7:**
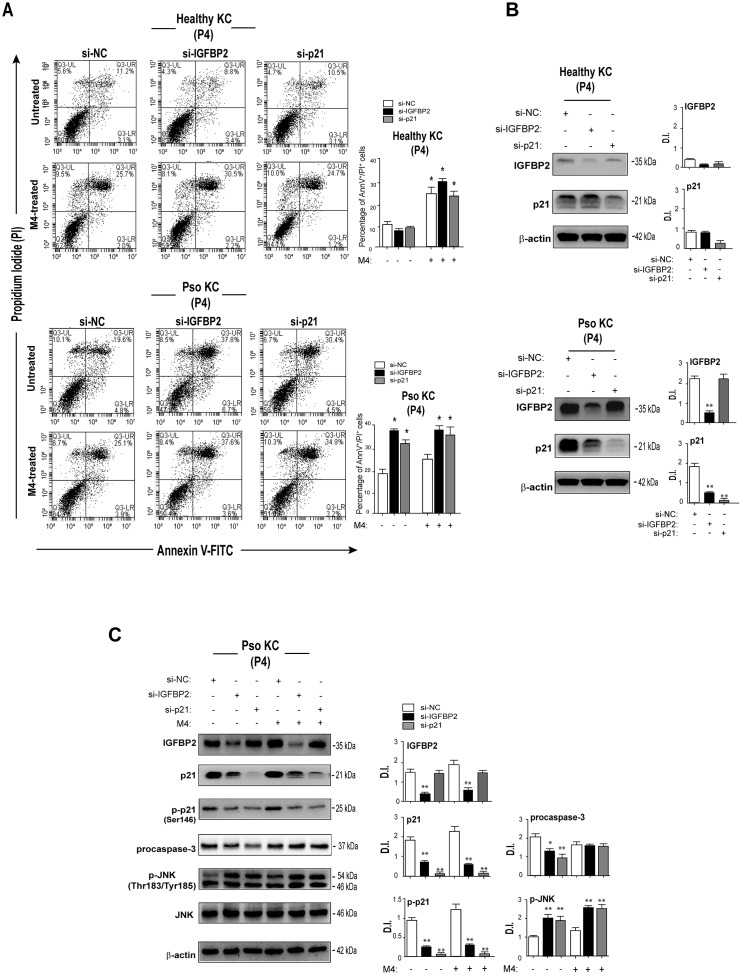
**IGFBP2 suppression leads to enhanced apoptosis of senescent psoriatic keratinocytes.** (**A**) Pso KC and healthy KC cultures at passage P4 were silenced or not for IGFBP2 or p21 for 48 hours, and treated or not with M4 for 24 hours. Apoptosis was examined by measuring Annexin V/PI fluorescent staining through FACS analysis. Graphs show the mean ± SD of the percentage of AnnV/PI double-positive cells of three independent experiments. *p ≤ 0.05, as calculated by paired Student’s *t* test comparing si-IGFBP2 or si-p21 with si-NC, or untreated with M4. (**B**) Protein lysates of untreated healthy KC and pso KC, silenced or not for IGFBP2 or p21, were analysed by WB to confirm IGFBP2 or p21 silencing. Graphs show the mean ± SD of densitometric intensity (D.I.) of three independent experiments. *p ≤ 0.05, **p ≤ 0.01 as calculated by paired Student’s *t* test comparing si-IGFBP2 or si-p21 with si-NC. (**C**) Protein extracts of pso KC at passage 4, silenced or not for IGFBP2, or p21 for 48 hours, and stimulated or not with M4 for 24 hours, were subjected to WB for the detection of IGFBP2, p21, p-p21, procaspase 3, p-JNK and JNK protein expression. WB panels are representative of three independent experiments and graphs show the mean ± SD of densitometric intensity (D.I.) of three independent experiments. *p ≤ 0.05, **p ≤ 0.01 as calculated by paired Student’s *t* test comparing si-IGFBP2 or si-p21 with si-NC.

Previous reports showed that the reduced susceptibility to apoptosis is a key feature of senescent keratinocytes, whose proliferation is arrested following confluency or exposure to anti-proliferative agents, such as IFN- γ [31]. In line with these studies, we previously demonstrated that psoriatic keratinocytes are less susceptible to cytokine-induced apoptosis, compared to healthy cells, although this feature was not explored in the context of senescence [8]. To investigate on the potential involvement of IGFBP2 in the mechanisms concurring to the cell death resistance of psoriatic keratinocytes, apoptosis was evaluated in cultures of senescent pso KC transiently silenced for IGFBP2 and compared to that observed in healthy KC, by AnnexinV (AnnV) staining and PI incorporation. As shown in Figure 7A, accordingly to our previous findings [8], M4 cytokine stimuli did not determine a significant induction of apoptosis in pso KC, compared to healthy cells, evaluated in terms of percentage of AnnV^+^/PI^+^ cells. Of note, IGFBP2 abrogation in pso KC cultured at passage P4, mainly characterized by very large, senescent cells (~50%), treated or not with M4, led to an increased percentage of apoptotic AnnV/PI double-positive in both untreated and cytokine-stimulated cells (Figure 7A, lower panels). Interestingly, similarly to IGFBP2, the silencing of p21 in senescent Pso KC cultures resulted in an increase of percentage of apoptotic keratinocytes, in both pso KC groups (Figure 7A, lower panels). In contrast, neither IGFBP2 or p21 silencing affected the viability of healthy KC cultures (Figure 7A, upper panels), expressing lower levels of IGFBP2 and p21 compared to pso KC (Figure 7B).

At molecular level, IGFBP2 and, at higher extent, p21 knock-down determined a decrease of inactive procaspase 3 levels in the untreated pso KC, but not in M4-treated cells, thus suggesting its processing in the active form. Unfortunately, the active caspase 3 form (~17 kDa) was not detectable by WB, although two different antibodies were used. Vice versa, IGFBP2 or p21 abrogation led to a marked increase of the phosphorylated levels of the pro-apoptotic JNK in both untreated and M4-treated senescent pso KC (Figure 7C).

Another feature of the senescence state is the SASP activation, characterized by the release of a set of inflammatory molecules acting in an autocrine loop on growth arrest of keratinocytes. IGFBP2 knock-down did not affect the basal and M4-induced production of IL-1β, IL-6, CXCL8, CCL20 and CCL2 compared to control-silenced cells (Supplementary Figure 5), thus excluding IGFBP2 involvement in the pro-inflammatory responses associated with senescence state.

In aggregate, these results demonstrated that IGFBP2 contributes to the arrest of proliferation and to the resistance to apoptosis typically exhibited by senescent psoriatic keratinocytes, but not to SASP activation.

## DISCUSSION

In this study, we describe for the first time the intracellular role of IGFBP2 in keratinocytes of skin affected by plaque psoriasis, known to be characterized by hyper-proliferation, impaired terminal differentiation and marked senescence changes.

The interest toward IGFBP2 in the psoriatic context arises from the observation that keratinocytes cultured from the lesional skin of psoriatic patients displayed increased expression of IGFBP2 already at the first culture passage, characterized by a subpopulation of small, proliferating cells and by a discrete number of senescent p16-positive cells. During senescence progression, these cultures rapidly enriched in p16 positive cells, in concomitance with prominently increased expression of IGFBP2. We found that in the subpopulation of small, proliferating cells, modestly present in the first culture passages of psoriatic cultures, IGFBP2, together with p21, was located in the nuclear compartment. Indeed, IGFBP2 exhibits a nuclear localization signal motif, responsible for its nuclear translocation, and, in cancer cells, nuclear IGFBP2 is involved in the transcriptional activation of the VEGF-A gene [26, 29]. The nuclear function of IGFBP2 in pre-senescent psoriatic keratinocytes remains undefined. In contrast, in senescent cells of psoriatic cultures, IGFBP2 was mainly localized in the cytoplasmic compartment, where p16 and, notably, phosphorylated p21 also accumulated. We showed an expected increase of p16 expression during senescence in both normal and psoriatic keratinocytes *in vitro* [58, 30, 31, 32]. In contrast, the expression of the cell-cycle inhibitor p21, together with its phosphorylated form, progressively diminished during senescence of both groups, although its levels remained higher in psoriatic cultures than those observed in normal cells at all stages of senescence progression. The decline of p21 observed in our *in vitro* models of senescence are consistent with an already demonstrated progressive decline of p53, a pivotal transcriptional regulator of p21, during the replicative senescence of normal keratinocytes [33, 34]. We suppose that, other than p21, the levels of the oncosuppressor p53 also remain sustained during senescence of psoriatic keratinocytes, and this feature could contribute to their reduced susceptibility to transform into malignant cells [35]. This hypothesis is supported by previous reports demonstrating a strong accumulation of p53 in the epidermis of lesional skin of patients affected by plaque psoriasis [36, 37].

Of note, the decline of p21 was not observed in pso KC upon stimulation of Th1/17-related cytokines. Indeed, the combination of pro-inflammatory cytokines augmented p21 protein, but not mRNA, levels in senescent pso KC, whereas it did not influence its amounts in healthy KC. We can speculate that, likely due to their genetic background, pso KC aberrantly respond to pro-inflammatory cytokines, by inducing molecules involved in senescence state, including p21 and IGFBP2.

In support of this hypothesis, in contrast with healthy skin, we observed a weak *in vivo* expression of IGFBP2 also in non lesional skin of psoriatic patients. This expression, together with that weakly observed for p21, could be the consequence of previous exposure of the skin biopsies examined in this study to pro-senescence stimuli or could be due to intrinsic genetic factors of the psoriatic patients. This last hypothesis seems to be supported by our observation that the over-expression of exogenous IGFBP2 in normal human keratinocytes did not trigger senescence changes, suggesting that other IGFBP2-independent molecular events must be necessarily implicated in the senescence switch of psoriatic cells.

As aforementioned, p16 and p21 are two senescence markers involved in the arrest of cell cycle. Differently from p16, p21 can play distinct actions, depending on its subcellular localization [20]. In the nucleus, p21 sustains the arrest of cell cycle, by inhibiting the activity of Cdk1 and Cdk2, and thus blocking the transition from G1 into S phase. In the cytoplasmic compartment, p21, following phosphorylation, has an anti-apoptotic action, by binding and inhibiting caspase 3, as well as the apoptotic kinases ASK1 and JNK [22].

**Figure 8:**
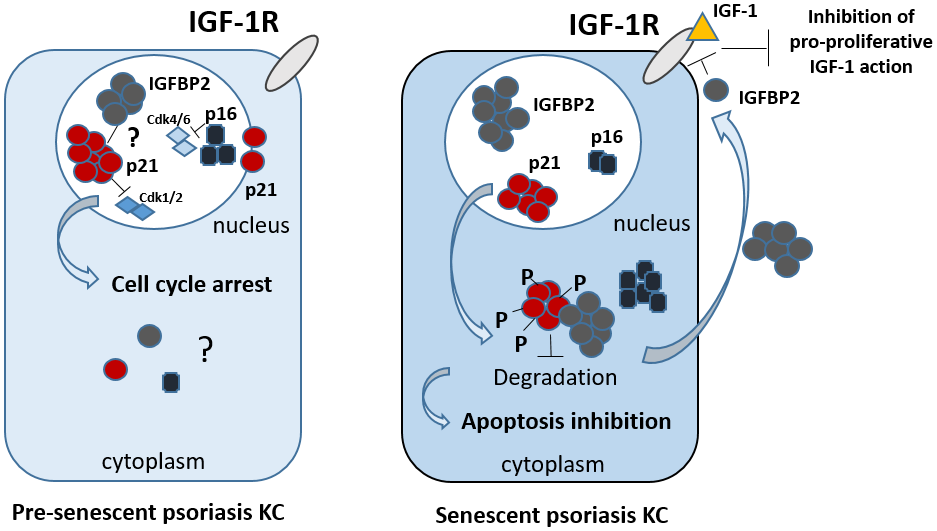
**Proposed integrated model of the intracellular and extracellular functions of IGFBP2 in psoriasis keratinocytes.** In epidermal keratinocytes of psoriasis patients characterized by a pre-senescent state, IGFBP2, p21 and p16 co-localize in the nuclear compartment. Here, p21 and p16 contribute to the cell cycle arrest in phase G1, by inhibiting Cdk1/2 and Cdk4/6, respectively. The nuclear role of IGFBP2 in pre-senescent KC remains to be investigated. In senescent psoriasis KC, IGFBP2, p16 and, of note, p21 accumulate in the cytoplasm. Here, p21 is hyper-phosphorylated and inhibits apoptotic processes. In the cytoplasm, IGFBP2 physically interacts with p21 and protects it from proteasomal degradation, thus sustaining its levels, and, indirectly, contributing to the apoptosis resistance typical of affected KC. In parallel, IGFBP2 released extracellularly blocks the pro-proliferative action of IGFs by binding to it and impeding its recruitment to IGF receptor.

We provide evidence that the unphosphorylated form of p21 accumulated *in vivo* in the nuclear compartment of keratinocytes located in the suprabasal, senescent layers of psoriatic lesions, and this is a typical feature of senescent cells, as recently demonstrated [19]. Interestingly, in line with previous reports, we found that phosphorylated p21 (Ser146) was mainly distributed in the cytoplasm of senescent cells, where it co-localized with IGFBP2 (Figure 8). Furthermore, we demonstrated that in senescent psoriatic keratinocytes, IGFBP2 sustains p21 protein accumulation and concomitant upregulation of p27, another member of the same class of cyclin-dependent kinase inhibitors involved in senescence [38, 39]. A more detailed analysis revealed that IGFBP2 physically interacted with the labile p21 protein in senescent cells, thus protecting it from ubiquitin-dependent proteasomal degradation. Thus, this interaction functionally resulted in the stabilization of the cytoplasmic levels of p21 (Figure 8). Our findings are in line with a previous study showing the physical interaction between IGFBP2 and p21 in lung epithelial growth-inhibited cells [27]. However, IGFBP2 is not the only protein stabilizing p21, since multiple p21 binding proteins, such as WISp39, Nucleophosmin/B23 and hSSB1, have been identified as companions or chaperones able to protect p21 from proteolytic cleavage [40–42].

Furthermore, a more detailed functional characterization unveiled that IGFBP2 sustained anti-apoptotic programs in senescent psoriatic keratinocytes, known to be less susceptible to UV- or cytokine-induced apoptosis compared to healthy cells [8, 43]. Indeed, the abrogation of p21, as well as that of IGFBP2, in senescent psoriatic keratinocytes resulted in an increase of apoptosis in both steady-state and cytokine treatment conditions, and it was caused, at least in part, by the intracellular activation of caspase 3 and of JNK protein, this last known to trigger pro-apoptotic mechanisms in human keratinocytes, and in particular, in the psoriasis context [44]. In contrast, healthy keratinocytes undergoing senescence were characterized by low p21 and IGFBP2 levels, and resulted to be more susceptible to the pro-apoptotic action of psoriasis-related inflammatory cytokines. In addition, their basal or cytokine-induced apoptosis was unaffected by p21 or IGFBP2 abrogation.

In addition, IGFBP2 knock-down resulted in an enhancement of the proliferative potential of pre-senescent psoriatic keratinocytes, where p21 was found to have a nuclear localization. We can speculate that in this subpopulation, IGFBP2 sustained the nuclear functions of p21, which, as aforementioned, are linked to the cell cycle arrest in different cell types (Figure 8).

In the pre-senescent state of epithelial cells, characterized by low levels of p16, p21-dependent cell cycle arrest is a reversible event [33, 45]. In fact, although both p21 and p16 are associated with the block of cell cycle, their pathways can either antagonize or synergize in senescence, depending on the type and level of stress, as well as on their respective levels. During senescence processes, several stressors may chronically act in concert to engage these effector pathways and induce different senescent phenotypes. Cells exposed to moderate chronic stresses may retain their proliferative potential extending cell cycle duration in a p21-dependent manner to allow a compensatory repair mechanism and restore proliferation. However, severe damages induce sustained activation of p21 and/or p16 pathways that execute full senescence process by triggering extensive chromatin remodelling [46, 47].

In light of these notions, we can suggest that IGFBP2 might contributes to senescence progression, by favouring an early p21-mediated cell cycle arrest in the nucleus that may then progress into full senescence following p16 upregulation. In the late stages of senescence, IGFBP2 expression could be upregulated by p16 itself, as demonstrated in this study, suggesting that p16-dependent chromatin remodelling may further promote IGFBP2 expression [48] (Figure 8).

We demonstrated also that *in vivo* IGFBP2 is highly expressed in the senescent keratinocyte compartment of psoriatic plaques, which is confined to the upper layers of epidermis and characterized by a strong expression of senescent markers, such as p21 and p16, suggesting its plausible involvement in the biological processes leading to the formation of the psoriatic plaque. In these lesions, keratinocyte senescence reasonably represents a stress response to aberrant cell division and concomitant defective differentiation and is thought to protect psoriatic lesions from tumorigenesis [49]. By contrast, the presence of a robust senescent compartment could be implicated in the perpetuation of the local inflammatory processes typical of psoriasis, through the release of pro-inflammatory mediators, including cytokines, chemokines and extracellular matrix proteolytic enzymes [7]. In parallel, the senescence state of the epidermal keratinocytes could contribute to epidermal thickening by sustaining anti-apoptotic programs and thus impeding the homeostatic mechanisms of epidermal renewal. In this last context, IGFBP2 could act as a pivotal mediator of the reduced susceptibility to apoptosis and the impaired terminal differentiation exhibited by psoriatic keratinocytes.

Besides having an important intracellular function, IGFBP2 acted extracellularly by contrasting the pro-proliferative action of IGF-1, in part mediated by CXCL8 release (Figure 8). IGF-1 contributes to epidermal hyperplasia of psoriatic skin lesions, by promoting proliferation of keratinocytes and protecting them from abnormal maturation and differentiation [50–53]. In contrast to IGFBP2, IGFBP3 has been reported to be essentially localized in the proliferative compartment of the psoriatic epidermis, with a peculiar accentuation in the suprapapillary epidermis where Ki67-positive proliferating cells were poorly represented [54]. To date, its function in psoriatic context remains unexplored. We can speculate that, while extracellular IGFBP2 inhibits the mitogenic action of IGF-1 on keratinocytes, in the attempt to reduce the epidermal hyperplasia, intracellular IGFBP2 contributes to a senescent switch of psoriatic cells, peculiarly resistant to apoptosis. The concomitant impairment in the processes of terminal differentiation and cornification, also due to the local inflammatory microenvironment, could definitively leads to the epidermal thickening and plaque formation. Further studies will be needed to define the mechanisms underlying IGFBP2 upregulation in pre-senescent and senescent psoriatic keratinocytes, and to clarify the nuclear function of IGFBP2 in the pre-senescence keratinocytes.

## MATERIALS AND METHODS

### Human subjects

Ten patients (aged 40-70 years) with mild-to-severe chronic plaque psoriasis (4 ≤ Psoriasis area and severity index ≥ 45) were included in this study. Biopsies were taken from skin plaques at sites overlapping lesional (LS) and the adjacent pre-lesional (Pre-LS), as well as at non-lesional sites (NLS), 3 cm distant from the developing plaques, all from the same psoriatic patients. In parallel, skin biopsies were also taken from six healthy volunteers undergoing plastic surgery and six from patients with chronic atopic dermatitis (AD). All individuals were analysed for immunohistochemical and for RT-PCR studies. Keratinocytes cultures were obtained from 4-mm biopsies taken from the LS and NLS skin of patients with chronic psoriasis (*n* = 6) and from normal skin of healthy subjects (*n* = 6).

This study was approved by the Ethical Committee of the IDI-IRCCS Hospital, Rome (registration no.: IDI-IMM-IL36pso) and performed accordingly to the Declaration of Helsinki. Informed consent was signed by all study subjects.

### Keratinocyte cultures and treatments

Human keratinocytes were established from sun-protected skin of healthy individuals (healthy KC) or psoriatic (pso KC) skin biopsies and cultured as previously reported [55, 56]. Cells were seeded (1.2-2x10^4^/cm^2^) on a feeder layer of irradiated 3T3 fibroblasts and cultured as previously described [57]. For serial propagation, starting from passage P0, cells were passaged at the stage of subconfluence, until they reached the senescence state (starting from passage P4) [58]. For a set of experiments, keratinocytes were stimulated with a mixture of four inflammatory cytokines (M4), including recombinant human IFN-γ (200 U/ml), TNF-α (50 ng/ml), IL-22 (50 ng/ml) and IL-17A (50 ng/ml) (R&D Systems, Minneapolis, MN, USA) in keratinocyte basal medium KBM Clonetics (San Diego, CA, USA), for different times as specified in the figure legends. When necessary, keratinocyte cultures were stimulated with IGF-1 or IGF-2 (10 ng/ml, R&D Systems) or an IGFBP2 blocking antibody (1μg/ml R&D Systems) or treated with MG132 (20 μM final concentration, Sigma-Aldrich, St. Louis, Missouri, USA) for 6 hours for the ubiquitination assays.

### RNA isolation and real-time RT-PCR

Total RNA was extracted from keratinocyte cultures and human skin biopsies, by using the TRIzol reagent (Invitrogen, Carlsbad, CA, USA) and RNeasy Lipid Tissue Kit (Qiagen, Chatsworth, CA, USA), respectively. mRNA was reverse-transcribed into complementary DNA by using SuperScript IV VILO reaction master mix (Invitrogen) and analysed by real-time PCR. GAPDH or β-actin were used as housekeeping genes, as specified in the Figure legends. Primer pairs used in PCR reactions are listed in the table reported in Supplementary Table 1. Fluorescence intensity was analysed by the ABI PRISM SDS 7000 PCR Instrument (Applied Biosystems, Branchburg, NJ, USA), using SYBR Green PCR reagents or Taqman PCR Master Mix. The values obtained from triplicate experiments were averaged, and data are presented as means of 2^-DDCT values ± SD.

### Immunoprecipitation, immunoblotting and densitometry

Total protein extracts were obtained in RIPA lysis buffer or, in case of co-immunoprecipitation, in EIA lysis buffer [250 mM NaCl, 50 mM Hepes (pH 7.5), 0.1% NP-40, 5 mM EDTA], protease inhibitor cocktail (Roche, Basel, Switzerland), and phosphatase inhibitor cocktail (Sigma-Aldrich), as previously reported [59]. Alternatively, cytosolic and nuclear extracts were prepared from cells grown in 75-mm flasks, which were lysed with 1 ml cold Buffer A (20 mM HEPES [pH 7.9], 10 mM KCl, 1 mM EDTA, 1 mM EGTA, 1.5 mM MgCl2, 0.2% NP-40, and 1 mM DTT plus protease and phosphatase inhibitors). After centrifugation, the supernatants containing cytoplasms were collected, whereas the pellets containing nuclei were resuspended in 0.4 ml cold Buffer B (20 mM HEPES [pH 7.9], 0.35 M NaCl, 10 mM KCl, 1 mM EDTA, 1 mM EGTA, 1.5 mM MgCl2, 10% glycerol, and 1 mM DTT plus protease and phosphatase inhibitors). After incubation at 4°C for 30 min, the suspensions were centrifuged at 14,000 rpm for 10 min, and the supernatants collected and diluted 5-fold in Buffer C (20 mM HEPES [pH 7.9], 60 mM NaCl, 10 mM KCl, 1 mM EDTA, 1 mM EGTA, 1.5 mM MgCl2, 5% glycerol, 0.05% NP-40, and 1 mM DTT plus protease and phosphatase inhibitors) [60]. The resulting samples were aliquoted and frozen at –80°C. For immunoprecipitation, lysates were precleared with immunoglobulin G (IgG) and then incubated with the precipitating antibody overnight, followed by 1 hour of incubation with protein A/G beads (Santa Cruz Biotechnology, CA, USA). Immune complexes were then washed three times in lysis buffer and boiled in gel loading buffer. Proteins from total, nuclear, cytosolic lysates or immunoprecipitates were resolved by SDS-polyacrylamide gel electrophoresis, transferred to nitrocellulose membrane, blocked in 5% not-fat milk or bovine serum albumin, and blotted with the appropriate antibody. Antibodies used were as follows: anti-p16 (sc-467), -p21 (sc-6246), -phospho-p21 (Ser146, sc-377515), -IGFBP2 (sc-6001), -IGFBP3 (sc-9028), -PCNA (sc-56), -p27 (sc-528), -p53 (sc-126), -p-Rb, -cyclin D (sc-8396), cdk1 (sc-53219), cyclin A (sc-751), -β-actin (sc-1615), -α-tubulin (sc-5286) and -lamin A/C (sc-7293), all purchased from Santa Cruz Biotechnology. Anti-phospho-Rb (Ser807/811, 9308), -ubiquitin (43124), -procaspase 3 (96625), -phospho-SAPK/JNK (Thr183/Tyr185, 92151), and -SAPK/JNK (9252) Abs were provided from Cell Signaling Technology (Danvers, Massachusetts, USA). As secondary antibodies, anti-mouse, anti-goat, or anti-rabbit Ig Abs conjugated to horseradish peroxidase were accordingly used and detected by the ECL-plus detection system (Amersham, Dubendorf, Switzerland), or, otherwise, the SuperSignal West Femto kit (Pierce, Rockford, IL, USA), by using an Imaging Densitometer GS- 670 (Bio-Rad, Hercules, CA, USA). Densitometric analyses were performed using Image J software (https://imagej.nih.gov/ij/), and band intensities were detected in three independent experiments and reported as means of Densitometric Intensity (D.I.) ± SD.

### Immunohistochemistry

Paraffin-embedded sections were obtained from biopsies of psoriatic skin including LS, Pre-LS and NLS zones of evolving plaques, as well as of healthy skin. Paraffin-embedded sections obtained from patients affected by AD were retrieved by Histopathology Unit at IDI-IRCCS hospital.

5-μm paraffin-embedded sections were dewaxed and rehydrated. After quenching endogenous peroxidase, achieving antigen retrieval, and blocking nonspecific binding sites, sections were incubated with mAbs against IGFBP2 (sc-6001; 1:40 dilution), p16 (sc-467; 1:20 dilution), p21 (sc-6246; 1:50 dilution), and p-p21 (sc-377515; 1:100 dilution), all purchased from Santa Cruz Biotechnology. Secondary biotinylated mAbs and staining kits were obtained from Vector Laboratories (Burlingame, CA, USA). Immunoreactivity was revealed using avidin-biotin-peroxidase system and 3-amino-9-ethylcarbazole as chromogen. Sections were counterstained with Mayer’s hematoxylin. As negative control, primary Abs were omitted or replaced with an irrelevant isotype-matched mAb.

A semiquantitative, four-stage scoring system was performed with the automated Image J software. The grading of the epidermal staining score ranged from negative immunoreactivity (0) to strong immunoreactivity (4+) for the evaluation of IGFBP2, p16 and phospho-p21expression. p21-positive cells were counted blindly by two observers with an eyepiece graticule at 200x magnification. For each skin specimen, two sections were analysed for each staining, and the positivity was calculated in five adjacent fields.

### Neutralization of IGFBP2 activity

To investigate the bioactivity of IGFBP2 released by psoriatic keratinocytes, cell supernatants were first 10-fold concentrated in 3,000 Da molecular weight cut-off concentrators (Vivascience, Hannover, Germany) and then extensively dialyzed against complete medium (KGM) in 20,000 Da molecular weight cut-off membranes (Spectrum Laboratories Inc., Los Angeles, CA). For the specific neutralization of IGFBP2 bioactivity in keratinocyte supernatants, the neutralizing goat polyclonal anti-human IGFBP2 Ab (AF674) or goat IgG control (AB-108-C) from R&D Systems was used. The exact dose of anti-IGFBP2 Ab to obtain effective IGFBP2 neutralization was set in preliminary experiments, and, in our conditions, corresponded to a 20-fold higher concentration (expressed in ng/ml) of the Ab respect to the concentration of IGFBP2.

### 5-Bromo-2-deoxy-uridine (BrdU) staining

Immunocytochemical assay for detection of BrdU after its incorporation into cellular DNA was performed on cells grown on coverslips with a dedicated kit Thermo Fisher Scientific (Waltham, Massachusetts, USA). Briefly, cells were labelled for 24 hours with KGM supplemented with 10 μM BrdU. They were then fixed with 4% (w/v) paraformaldehyde (PFA) in PBS for 10 min at room temperature, and permeabilized with 0.05% Triton X-100 in PBS containing 1% BSA. Finally, after a DNA hydrolysis step, BrdU was immunostained with a biotinylated anti-BrdU Ab visualized with DAB-peroxidase substrate. BrdU-positive cells were counted in high power fields and expressed as cells per area unit ± SD (n [microscopic fields per slide] = 6).

### Immunocytochemistry

Cells were grown on chamber slides. After the indicated treatments, they were fixed with 4% paraformaldehyde in phosphate buffer saline (with calcium and magnesium salts, in order to avoid cell detachment) for 20 minutes at room temperature. Endogenous peroxidases were deactivated with 0.03% hydrogen peroxide for 10 minutes, and cell layers were then treated with Protein Block solution (Dako, Carpinteria, CA, USA) to reduce unspecific background. In order to allow intracellular antibody (Ab) diffusion, cells were permeabilized with 0.05% Triton X100 in Protein Block solution for 3 minutes. The anti-IGFBP2 primary Ab (sc-6001, Santa Cruz) was diluted in Antibody Diluent (Dako). A secondary biotinylated mAb and staining kits (from Vector Laboratories) were used to develop immunoreactivity, and 9-ethyl-3-aminocarbazole was used as substrate. Unless otherwise specified, cells were counterstained with hematoxylin. Negative controls followed the same treatments with the exclusion of the primary Ab, which was substituted with Antibody Diluent.

### Senescence-associated β-galactosidase activity assay

For the detection of senescence-associated β-galactosidase (SA-β-gal) activity, 4% (w/v) PFA-fixed keratinocyte monolayers grown in chamber slides were incubated (24 h, 37°C) in a staining solution containing β-galactosidase substrate X-gal (5-bromo-4-chloro-3-indolyl-β-D-galacto-pyranoside) in the presence of equimolar (5.0 mM) concentrations of potassium ferrocyanide and potassium ferricyanide [61]. All chemicals were purchased from Sigma Aldrich. The development of blue color was followed under a 200x magnification microscope.

### Immunofluorescence

Keratinocytes were seeded onto 24-wells containing 1.4-cm^2^ round glass coverslips and cultured as above. Cells were fixed with 4% PFA, and permeabilized with 0.1% Triton X-100. After blocking with 1% BSA, cells were incubated with goat polyclonal anti-IGFBP-2 Ab (sc-6001, Santa Cruz Biotechnology) and then with Alexa Fluor 488-conjugated anti-goat Ab. Subsequently, cells were incubated with mouse monoclonal anti-p21 (sc-6246, Santa Cruz Biotechnology), or p-p21 (Ser146, sc-377515, Santa Cruz Biotechnology) and then with Alexa Fluor 555 anti-mouse. Cells were also incubated with goat polyclonal anti-IGFBP-2 Ab (sc-6001, Santa Cruz Biotechnology) and then with Cy™3 Donkey anti-goat Ab (Jackson ImmunoResearch Laboratories, Inc. West Grove, PA, USA). Subsequently, cells were incubated with rabbit polyclonal anti-p16 (sc-467, Santa Cruz Biotechnology), and then with Alexa Fluor 488 anti-rabbit. The cells were counterstained with DAPI for the visualization of the nucleus. Images were captured with the ApoTome System connected with an Axiovert200 inverted microscope (Zeiss, Oberkochen, Germany). Image analysis was then performed with ZEN software (Zeiss).

### Retroviral-mediated gene transfer in primary keratinocytes

Stable keratinocyte transductions were carried out as previously described [58]. L(p16)SN [encoding the fusion protein p16INK4a-enhanced green fluorescent protein (EFGFP)] was constructed by cloning a 500 kb fragment containing the full-length human p16INK4a into the EGFP expression retroviral vector (LGSN-LoxP vector). L(AS-Exo1a)SN was constructed by generating a specific antisense fragment, corresponding to exon1a of p16INK4a gene that was cloned into the LXSN-LoxP retroviral vector. Am12L(p16) and Am12/L(as-Exo1a)SN packaging cell lines were generated by the infection protocol, as described previously [62].

Producer cell lines showed a viral titer of 0.5-1 x 10^6^ colony-forming units/ml. Control amphotropic packaging cell lines were generated as above, using LXSN-LoxP and LGSN-LoxP retroviral vectors.

### Transient RNA interference

IGFBP2 and p21 were knocked-down by using a pool of 4 small short interfering (si)RNA ON-TARGET plus SMARTpool, L-010896-00-0005 and L-003471-00-0005 respectively (Dharmacon RNA Technology, Lafayette, CO, USA). In parallel, a pool of four non-targeting siRNA (L-011511-00-0005) was used as negative control. Primary cultures of keratinocytes were transfected with IGFBP2-, p21- or control- siRNA at 50 nM final concentration, complexed to 4 μg/ml INTERFERin reagent (Polyplus Transfection, New York, NY, USA) for different times, as specified in the figure legends.

### Enzyme-linked immunosorbent assays (ELISA)

IGFBP2, IL-1β and CCL20 were measured with Duoset kits (R&D Systems), whereas and IL-6, CXCL8 and CCL2 levels were measured with OptEIATM kits (BD Pharmingen, Milan, Italy), in cell-free supernatants (sups) from resting or stimulated keratinocyte cultures, according to the manufacturer’s protocols. The plates were analysed in an ELISA reader mod.3550 UV Bio-Rad. Results are graphed as pg or ng/10^6^ cells ± SD.

### Keratinocyte proliferation

In total, 5×10^4^ IGFBP2- or NC-silenced keratinocytes were seeded in 12-well plates in KGM. 1day after, the cells were starved in KBM and treated with M4 cytokines, or left untreated, and cultured for 24 and 48h. The number of viable cells was determined by a Trypan blue exclusion test.

### Apoptosis analysis

Apoptosis of keratinocytes was evaluated using the FITC-AnnexinV/propidium iodide (PI) apoptosis detection kit (BD Pharmigen) and analysed by flow cytometry, by using a FACScan equipped with Cell Quest software. The percentage of Annexin V^+^, PI ^+^, and Annexin V/PI^+^ cell populations were evaluated in keratinocytes transiently silenced for IGFBP2 or p21 and in control cells, left untreated or treated with M4 cytokine stimulus.

### Transfection with IGFBP2 expression vectors

The pCMV-int-IGFBP-2 (kindly provided by Dr. Vincenzo C. Russo, University of Melbourne, Victoria, Australia) or the empty vector pcDNA3 were transfected into healthy keratinocytes, according to previous methods [60]. Briefly, keratinocytes were seeded onto 1.9 cm^2^ wells (2x10^5^ cells/well). At 60% confluence, cells were transfected over a 6 h period with lipofectin reagent (Life Technologies, Gaithersburg, MD, USA) and mixed with 0.5 μg of plasmids. Keratinocytes were then allowed to recover in fresh complete medium overnight, and then used for time-course experiments.

### Statistical analysis

Differences between groups were evaluated by the Mann–Whitney U or (unpaired or paired) Student’s t test, as specified in the figure legends, by using GraphPad prism Software (La Jolla, CA, USA). Significance was assumed at a *p* value of 0.05 or less.

## Supplementary Material

Supplementary Figures

Supplementary Table 1
