# Supplementary material for: Intracellular Insulin-like growth factor binding protein 2 (IGFBP2) contributes to the senescence of keratinocytes in psoriasis by stabilizing cytoplasmic p21
Source: Aging (Albany NY). 2020 Apr 17;12(8):6823–51. doi: 10.18632/aging.103045 (PMC7202509; doi:10.18632/aging.103045)
Supplement: Supplementary Figures [file aging-12-103045-s002..pdf]

SUPPLEMENTARY FIGURES

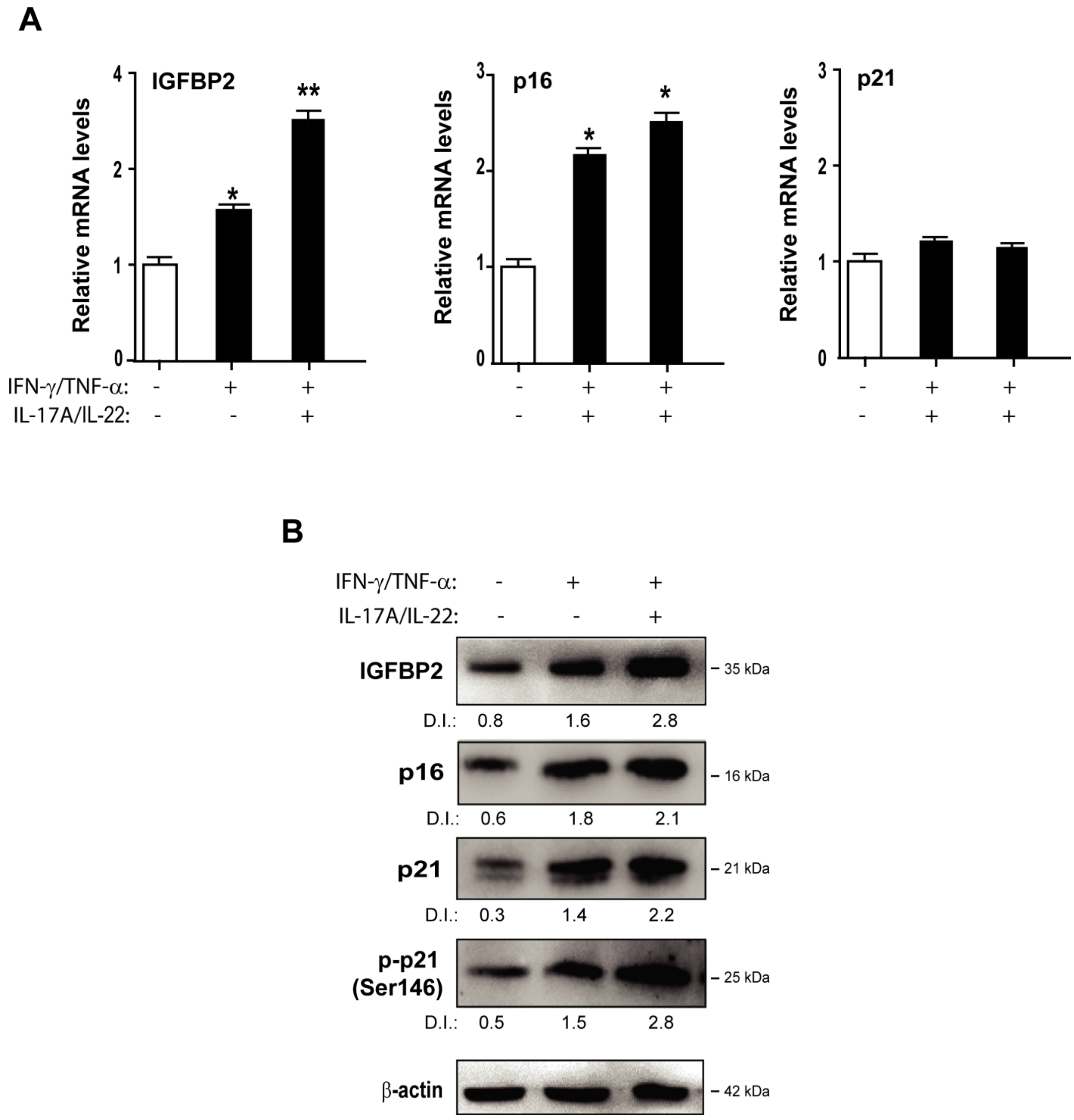

Supplementary Figure 1. IGFBP2 expression is upregulated by pro-inflammatory cytokines in psoriatic keratinocytes. (**A**, **B**) Transcriptional and protein levels of IGFBP2, p16 and p21 were detected by Real-time PCR analysis and WB, respectively, on keratinocytes isolated from NLS biopsies and cultured at passage P4. Cells were treated with IFN-γ and TNF-α, alone or in presence of IL-17A and IL-22 for 6 hours for transcriptional analysis, and for 18 hours for WB analysis. *$p \leq 0.05$, *$p \leq 0.01$, as assessed by paired Student's *t* test comparing untreated and cytokine-treated. The protein levels of phosphorylated p21 was also detected by WB. In (**B**), D.I. indicates mean values of densitometric intensity of each band obtained from three independent experiments.

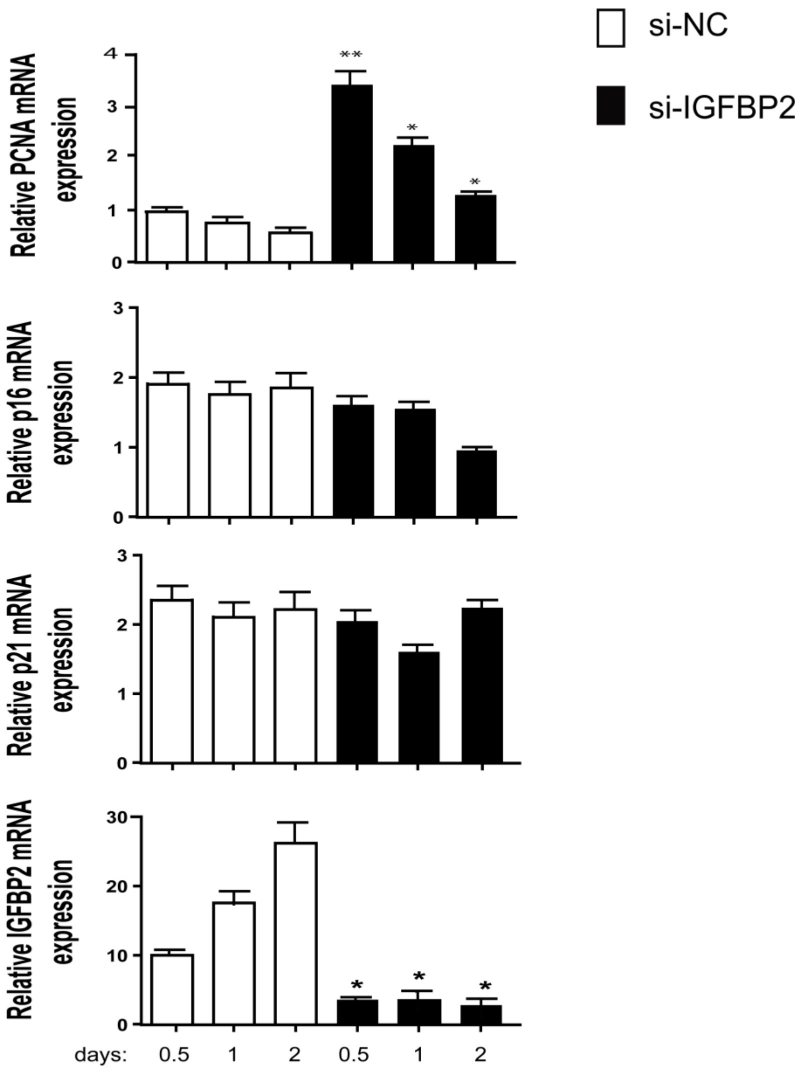

Supplementary Figure 2. IGFBP2 suppression does not influence transcriptional expression of p21 and p16, but induces PCNA mRNA expression. mRNA levels of PCNA, p16, p21 and IGFBP2 were detected by Real-time-PCR analysis on total RNA obtained from pso KC cultures (at P4 passage) transiently silenced for IGFBP2 (si-IGFBP2) or control cells (si-NC) at different time-points. Data shown are means of relative mRNA expression (normalized to β-actin) of three independent experiments. *$p \leq 0.05$, as assessed by paired Student's *t* test comparing si-IGFBP2 with si-NC groups at the same time-point.

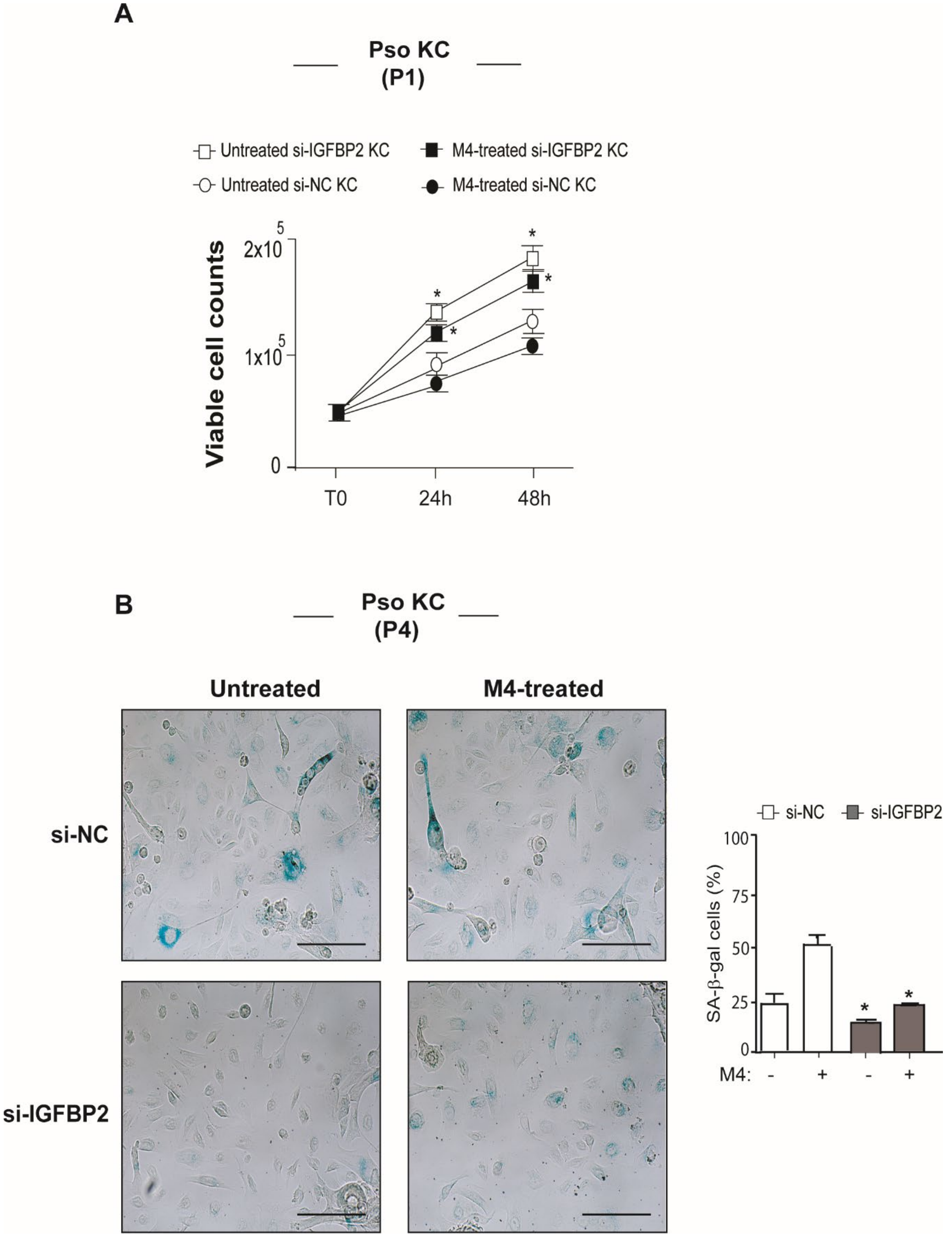

Supplementary Figure 3. IGFBP2 abrogation determines an enhanced proliferation and a reduced β-galactosidase activity in pso KC cultures. (**A**) Cell proliferation was evaluated in pso KC transiently transfected with NC- or IGFBP2-siRNA at passage P1, left untreated or M4-treated (24h and 48h after transfection), by using Trypan blue exclusion assay. (**B**) The activity of senescence-associated β-galactosidase (SA-β-gal) was detected by colorimetric staining (blue) in pso KC cultures at passage P4, silenced or not for IGFBP2, left untreated or M4-treated. Data are representative of three independent experiments. Bars, 100 µm. The graph shows the means of SA-β-gal positive cells ± SD, counted in two adjacent fields. *$p \leq 0.05$, as assessed by paired Student's *t* test comparing si-IGFBP2 with si-NC groups.

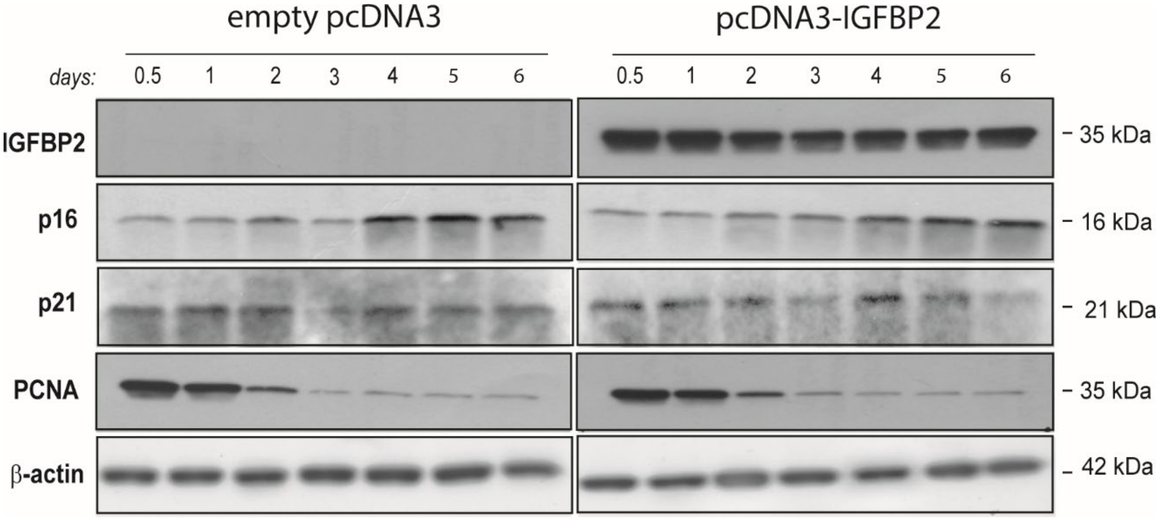

Supplementary Figure 4. IGFBP2 over-expression does not affect senescence markers in healthy keratinocytes. WB analysis of healthy KC at passage 2 (P2) transfected with the empty expression vector pcDNA3 (empty-pcDNA3, left panel) or with the vector bearing IGFBP2 (pcDNA3-IGFBP2, right panel) at different time-points, as indicated, to detect IGFBP2, p16, p21 and PCNA protein levels. Results showed are representative of three independent experiments.

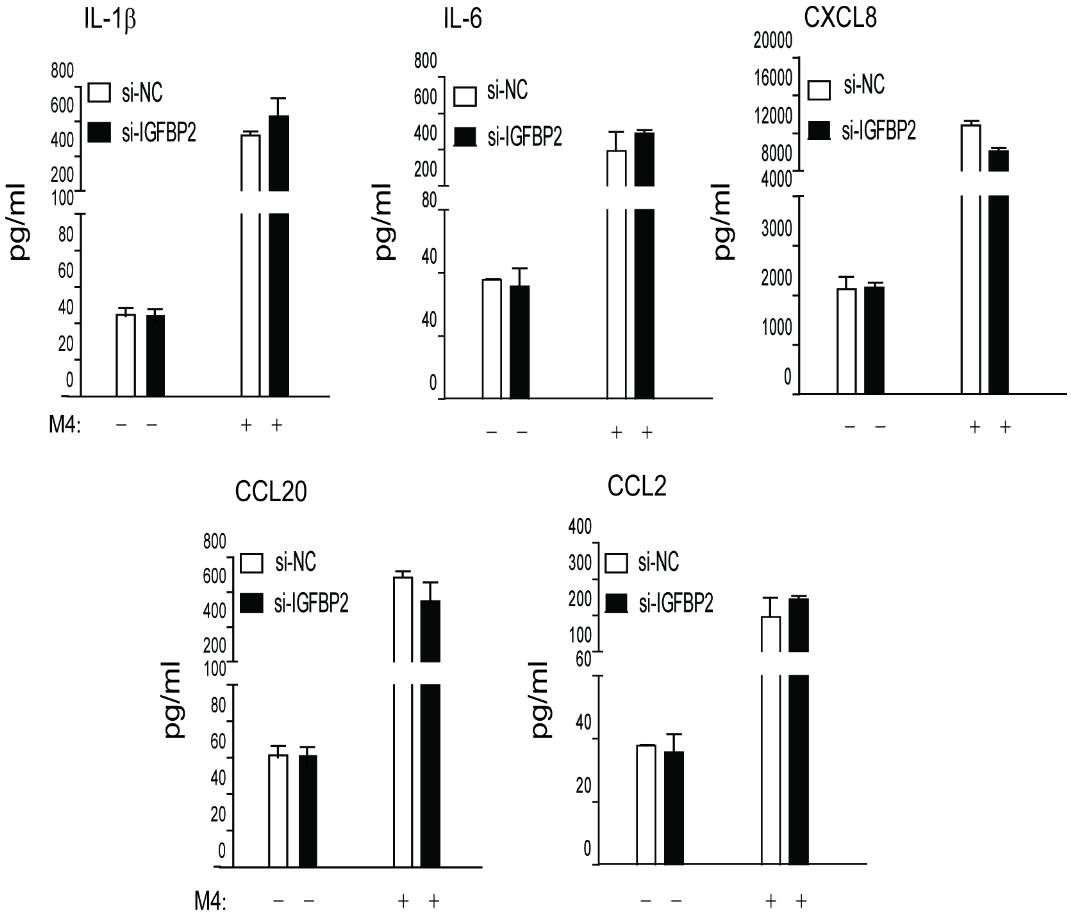

Supplementary Figure 5. IGFBP2 abrogation does not affect SASP in pso KC cultures. IL-1β, IL-6, CXCL8, CCL20 and CCL2 production was evaluated by ELISA on supernatants obtained from pso KC cultures silenced or not for IGFBP2 at passage P4 and stimulated or not for 24 h with M4. Results are expressed as means of pg/ml ± SD of three different experiments.
