# Supplementary material for: Intracellular Insulin-like growth factor binding protein 2 (IGFBP2) contributes to the senescence of keratinocytes in psoriasis by stabilizing cytoplasmic p21
Source: Aging (Albany NY). 2020 Apr 17;12(8):6823–51. doi: 10.18632/aging.103045 (PMC7202509; doi:10.18632/aging.103045)
Supplement: Supplementary Table 1 [file aging-12-103045-s001..pdf]

## SUPPLEMENTARY TABLE

**Supplementary Table 1. List of primers used for quantitative real-time PCR.**

| Gene | forward | reverse |
|---|---|---|
| IGFBP2 | GCCCGGAGCAGGTTGC | ACTGCCTCCCCCGCC |
| IGFBP3 | TGTGGCCATGACTGAGGAAA | TGCCAGACCTTCTTGGGTTT |
| p16 | CAACGCACCGAATAGTTACGG | CCACTCGGGCGCTGC |
| p21 | CAGGGTCGAAAACGGCG | GGCGTTTGGAGTGGTAGAAATC |
| Cdk1 | TTGAAAGCGAGGAAGAAGGA | CCCTGGAGGATTTGGTGTAA |
| Cyclin A | GCCATTAGTTTACCTGGACCCAGA | CACTGACATGGAAGACAGGAACCT |
| p57 | ACCTTCCCAGTACTAGTGCG | AAGTCGTAATCCCAGCGGTT |
| PCNA | TTGGCGCTAGTATTTGAAGCAC | TTCATAGTCTGAAACTTTCTCCTGGTT |
| CXCL-8 | CTCTGTGTGAAGGTGCAGTTTT | GGGTGGAAAGGTTTGGAGTAT |
| β-actin | TCCTGGGCATGGAGTCCTGT | TCGGCAATGCCAGGGTACAT |
| GAPDH | TGGACCTGACCTGCCGTCTA | CCCTGTTGCTGTAGCCAAATT |
